# Advancing sustainable agriculture: a critical review of smart and eco-friendly nanomaterial applications

**DOI:** 10.1186/s12951-023-02135-3

**Published:** 2023-10-11

**Authors:** Sri Renukadevi Balusamy, Abhayraj S. Joshi, Haribalan Perumalsamy, Ivan Mijakovic, Priyanka Singh

**Affiliations:** 1 Department of Food Science and Biotechnology, Sejong University, Gwangjin-Gu, Seoul, 05006 Republic of Korea; 2 The Novo Nordisk Foundation Center for Biosustainability, Technical University of Denmark, 2800 Kongens Lyngby, Denmark; 3 Institute for Next Generation Material Design, Hanyang University, Seoul, Republic of Korea; 4 Center for Creative Convergence Education, Hanyang University, Seoul, Republic of Korea; 5 Department of Chemistry, College of Natural Sciences, Hanyang University, Seoul, Republic of Korea; 6 Systems and Synthetic Biology Division, Department of Biology and Biological Engineering, Chalmers University of Technology, 412 96 Gothenburg, Sweden

**Keywords:** Sustainable agriculture, Green synthesis, Metal nanoparticles, Nanofertilizers, Foliar application, Seed priming

## Abstract

Undoubtedly, nanoparticles are one of the ideal choices for achieving challenges related to bio sensing, drug delivery, and biotechnological tools. After gaining success in biomedical research, scientists are exploring various types of nanoparticles for achieving sustainable agriculture. The active nanoparticles can be used as a direct source of micronutrients or as a delivery platform for delivering the bioactive agrochemicals to improve crop growth, crop yield, and crop quality. Till date, several reports have been published showing applications of nanotechnology in agriculture. For instance, several methods have been employed for application of nanoparticles; especially metal nanoparticles to improve agriculture. The physicochemical properties of nanoparticles such as core metal used to synthesize the nanoparticles, their size, shape, surface chemistry, and surface coatings affect crops, soil health, and crop-associated ecosystem. Therefore, selecting nanoparticles with appropriate physicochemical properties and applying them to agriculture via suitable method stands as smart option to achieve sustainable agriculture and improved plant performance. In presented review, we have compared various methods of nanoparticle application in plants and critically interpreted the significant differences to find out relatively safe and specific method for sustainable agricultural practice. Further, we have critically analyzed and discussed the different physicochemical properties of nanoparticles that have direct influence on plants in terms of nano safety and nanotoxicity. From literature review, we would like to point out that the implementation of smaller sized metal nanoparticles in low concentration via seed priming and foliar spray methods could be safer method for minimizing nanotoxicity, and for exhibiting better plant performance during stress and non-stressed conditions. Moreover, using nanomaterials for delivery of bioactive agrochemicals could pose as a smart alternative for conventional chemical fertilizers for achieving the safer and cleaner technology in sustainable agriculture. While reviewing all the available literature, we came across some serious drawbacks such as the lack of proper regulatory bodies to control the usage of nanomaterials and poor knowledge of the long-term impact on the ecosystem which need to be addressed in near future for comprehensive knowledge of applicability of green nanotechnology in agriculture.

## Introduction

**Fig.1:**
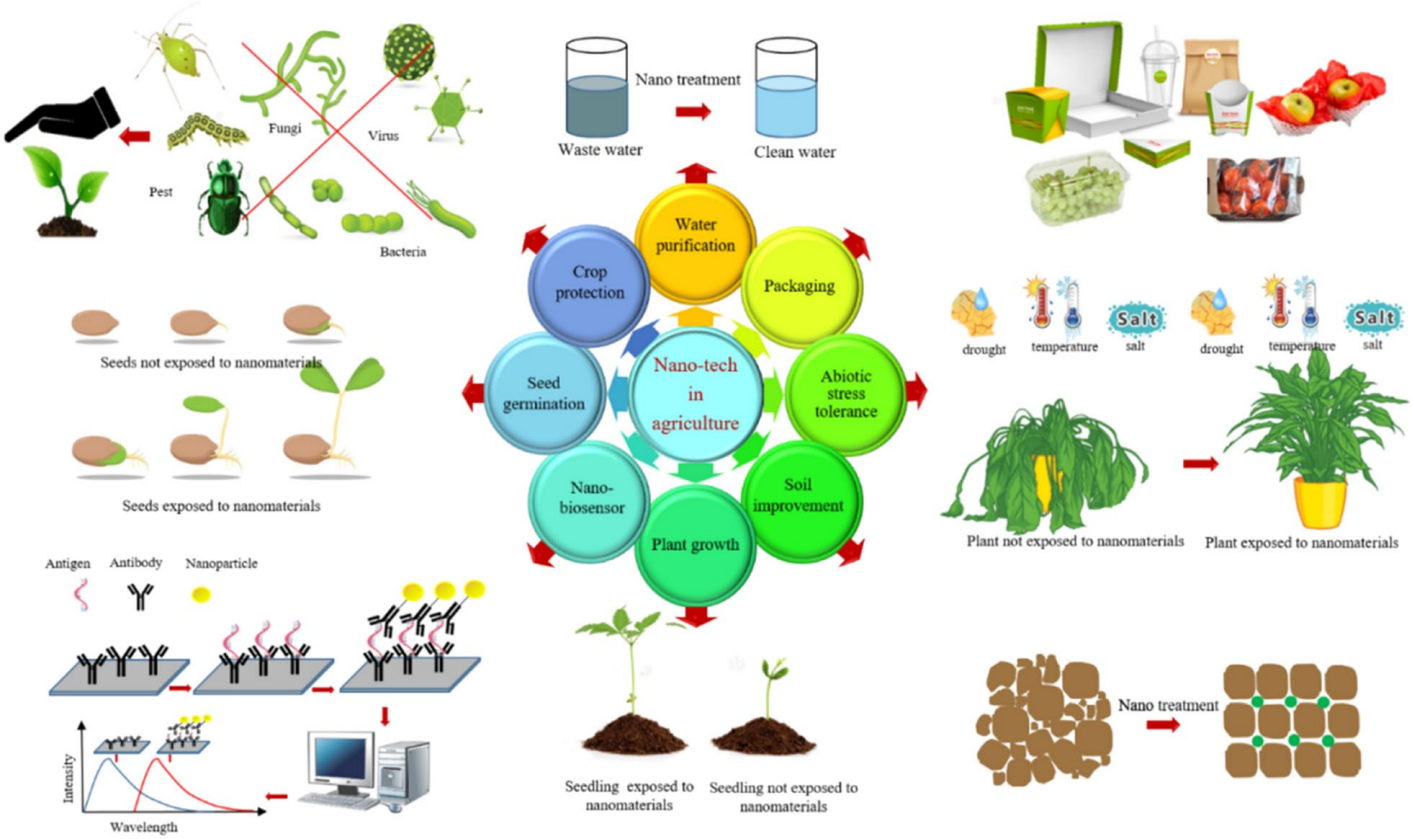
Application of nanomaterials in agriculture [5]. Copyright, 2022

Sustainability has become the core interest in the medical, industrial, and agriculture sectors. In agriculture, nanoparticles can also be used as nanofertilizers and nanopesticides [1]. Sustainable agriculture is the future, and to achieve it, scientists have developed various nanoparticles such as silver (AgNPs), gold (AuNPs), copper (CuNPs), zinc oxide (ZnONPs), and iron oxide (Fe3O4NPs) nanoparticles. Other nanomaterials (NMs) that play a critical role in achieving sustainability by improving efficiency and productivity in the agriculture sector are quantum dots (QDs), silica nanoparticles (SiNPs), carbon nanotubes (CNT), polymeric nanoparticles, and liposome-based NMs. The use of nanoparticles in agriculture showed following advantages **(**Fig. 1**)**. However, various types of methods exist for nanoparticle synthesis, green synthesis method offer ecofriendly and cheaper way for nanoparticle production. Synthesized nanoparticles constitute a smart technology with immense potential to revolutionize sustainable agriculture. Their application can lead to improved plant germination, enhanced growth, and increased tolerance to biotic and abiotic stresses, ensuring healthier crops and higher yields. Additionally, these nanoparticles contribute to improved soil health, fostering a sustainable and balanced ecosystem. Furthermore, their use offers increased safety for human consumption by reducing the presence of harmful chemicals, paving the way for a more environmentally friendly and sustainable agricultural system overall.

The final impact of nanoparticles on plants and associated ecosystem/biodiversity is not only governed by the precursors used for their synthesis and their size and/or shape; but also, by route of administration while applying in agricultural land, their surface coatings/surface functionalization, their chemical nature, and the payload that they deliver. Various methods of nanoparticle application in plants have been explored, each with its potential benefits and drawbacks. Seed nano-priming involves treating seeds with nanoparticle solutions before planting, which can positively influence germination and early growth stages [2]. Foliar spray application directly targets leaves, enabling efficient nutrient delivery and stress alleviation [3]. Soil mixture involves incorporating nanoparticles into the soil, offering prolonged nutrient release and improved soil health. Hydroponics, a soilless cultivation method, allows precise control of nutrient uptake by plants [4].

However, improper nanoparticle dosages or formulations can have negative effects on plants, such as toxicity or impaired growth. Therefore, careful consideration of nanoparticle application methods and dosage is essential to harness their full potential while mitigating any adverse impacts on plants. Therefore, knowing the suitable method of nanoparticle application depending on the nanoparticles used to plants is crucial for obtaining sustainable agriculture.

Apart from method of nanoparticle application, to improve effectiveness of NPs and to safely use them, NPs are coated with various chemical moieties as surface coatings of nanoparticles affects their physicochemical properties such as solubility, degradation, endocytosis as well as their fate in plant cells once entered. Vast literature is available that shows various types of coatings on surface of nanoparticles intended for agricultural use [6–8]. However, our review focuses on key articles with such topic and summarized their outcomes to comment on coatings and their positive and negative impacts on crops. Further, as an alternative approach of the use of fertilizers, pesticides, and herbicides, farmers adapt NPs to deliver essential components such as nutrients, fertilizers, antibiotics etc., by various methods such as seed priming, foliar spray, or soil mixture.

Despite growing volume of nanoparticle research in plants, the vast information on safe and toxic effects are sprinkled here and there and, hence, by deciding a correct way to synthesize nanomaterials using green technology, correct “safe method” of application of those nanomaterials to crops in fields, correct concentration that is optimal for beneficial effects on crops and soil with very low or zero nanotoxic effects, correct application methods that could minimize toxicity are need of the hour to build a suitable nanotechnological platform for precise and sustainable agriculture. For that purpose, it is essential to describe “safe methods” and “toxic methods” for the application of nanoparticles in plants, which form a bridge between their immediate impact on plant and soil health, plant development as well as long-term impact on ecosystem. Majority of previous reviews failed to address this issue. Our review article contributes significantly to this regard with the help of most recent research findings from various researchers across globe. This review could also help in making a decisive workflow for nanomaterials intended for agricultural use that starts from synthesis of nanomaterials and ends with their controlled usage in actual field by addressing the following topics (1) Green technology (2) Different routes of NPs application while applying in agricultural land (3) Their surface coatings/surface functionalization, their chemical nature, and the payload that they deliver (4) Use of NPs to deliver biomolecules to plants (5) Critical issues such as regulatory policies for controlling nanomaterial usage in agricultural land.

## Green synthesis

The application of nanoparticles (NPs) or nanomaterials (NMs) specifically depends on NP types, shape, size, and biological corona surrounding them. These properties further depend on the source of synthesis. Synthesis of nanoparticles can be achieved by following chemical, physical, and biological methodologies. Nevertheless, chemical and physical synthesis controlled and produce monodisperse NPs; there are a few limitations, such as the production of hazardous and toxic by-products, attachment of excessive chemicals on the surface of NPs. These limitations have led to the development of sustainable alternatives called green nanotechnology, where researchers mostly focus on the biological resources or green methodologies for the production of NPs. Green methodologies have been followed for more than a decade to synthesis several metal nanoparticles including gold and silver [9, 10]. The main purpose of green approaches is to enhance the NPs activity and reduce their impact on health and the environment. A tremendous amount of research has been done to find new green resources for NPs production and their possible outcomes. Green synthesis includes the synthesis of NPs using plants or their parts as well as using different microorganisms, including bacteria [11, 12], fungi [13], yeast, and viruses [14, 15]. Green synthesis has been appreciated as a quick facile and produces stable and biocompatible nanoparticles. Especially, medicinal plants are extensively reported for the rapid synthesis of nanoparticles ranging from a few seconds to a few hours, unlike bacteria synthesis, which often requires 24–48 h. So far, many medicinal plants have been reported to produce metallic nanoparticles, such as *Panax ginseng* [16], *Rhodiola rosea* [17]*, Cannabis sativa* [18], Rowan berries [19], Siberian ginseng, etc. Plant-mediated synthesis has been a promising way for nanoparticle mass production. It is eco-friendly, cost-effective, easily scaled up, and does not require high temperature or special energy resources (e.g., ultrasound waves). The components responsible for metal reductions in microorganisms are enzymes, proteins, and secondary metabolites, whereas in plants, those include flavonoids, terpenoids, phenols, carbohydrates, saponins, steroids, etc. The biological components mentioned above help in reduction and form a surrounding layer around the individual nanoparticles called the "capping layer" or "biological corona." The biological corona formed around the nanoparticles contains biological components released from the plant or microorganism in an extract or a culture medium used for synthesis. This capping layer offer the long-term stability of nanoparticles in aqueous solutions, protecting the nanoparticles from agglomeration, and most importantly, playing a major role in the interaction of green nanoparticles with cells [20]. This helps nanoparticles to permeate easily into the plant or bacterial or fungal cells and cell organelles. Thus, the biological corona plays key beneficial role for nanoparticle production and its applications in different fields.

### Factors influence the green synthesis

**Fig. 2:**
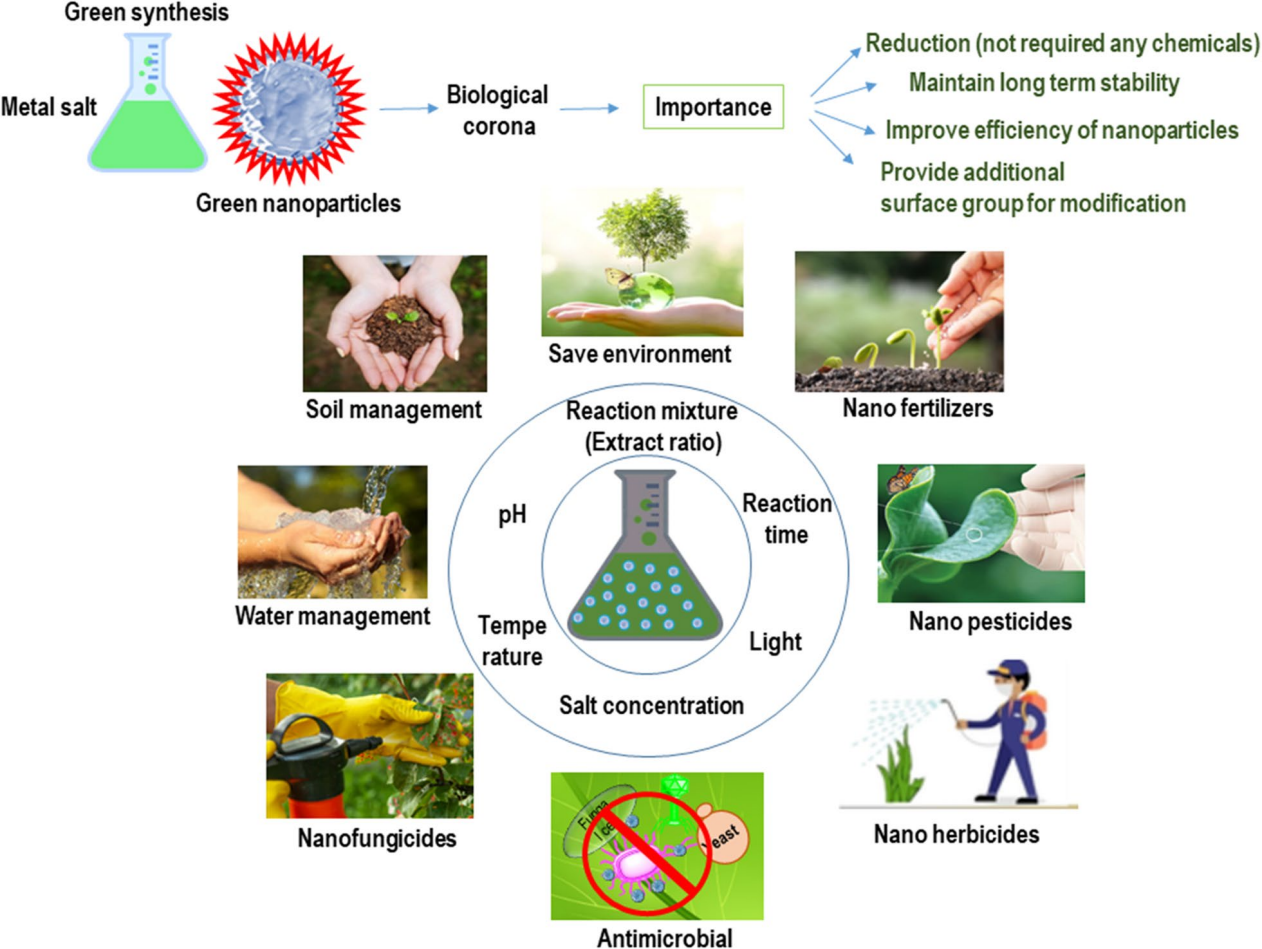
Green synthesis of nanoparticles, method parameters and applications in sustainable agriculture

The parameters that influence the metallic nanoparticle structure during synthesis are the source of synthesis, temperature, pH, salt concentrations, and time used for synthesis. Depending on these parameters, the metallic nanoparticles form and avail their activity (Fig. 2). Reaction time is crucial for nanoparticle synthesis, which decides nanoparticles’ shape, size, and stability. Singh et al. recently showed that an increase in the reaction time of synthesis of gold and silver nanoparticles leads to the whole agglomeration of nanoparticles at higher temperatures. At high temperature, 70–90 ℃, green synthesis occur very quickly [19]. However, the authors have noted that a long reaction time and higher temperatures cause agglomeration with different plant extracts. pH plays a major role in deciding nanoparticles’ size and shape. The literature suggests that big nanoparticles form fewer functional groups attached to the corona layer at acidic pH [21]. Effects of temperature on nanoparticle morphology have also been majorly investigated. The literature has evidenced that higher temperature helps in quick reduction and, if treated for a longer period, could cause agglomerations. Many biological components also become inactive at higher temperatures, which otherwise would have been available to attach to the nanoparticles under the corona. This causes the instability of nanoparticles. However, this is case-specific and does not imply all the green resources. Numerous reports have demonstrated stable, small, and monodisperse nanoparticles formed even at higher temperatures. For instance, Singh et al. showed the formation of the silver nanoparticles from Rowan berries at 90 ℃ temperature, which was 100% monodisperse in nature [19]. Another example demonstrated by Gericke et al. [22]. where spherical gold nanoparticles predominantly formed at a lower temperature. However, an increase in temperature allowed the change in the shape of nanoparticles and resulted in rod and plate-shaped nanoparticles [23]. The reaction components also affect nanoparticle formation, i.e., the biomolecules available for reduction. Huang et al. showed the change in the shape of gold and silver nanoparticles from triangular to spherical with an increasing concentration of sun-dried *Cinnamomum camphora* leaf extract [24]. The concentration-dependent behavior of *Aloe vera* leaf extract resulted in the alteration of gold nanoparticles’ shapes, transitioning them from spherical to triangular plates [25]. These examples highlight the significant role of reaction components in the synthesis of green nanoparticles.

### Factors influencing the toxicity of nanoparticles

**Fig. 3:**
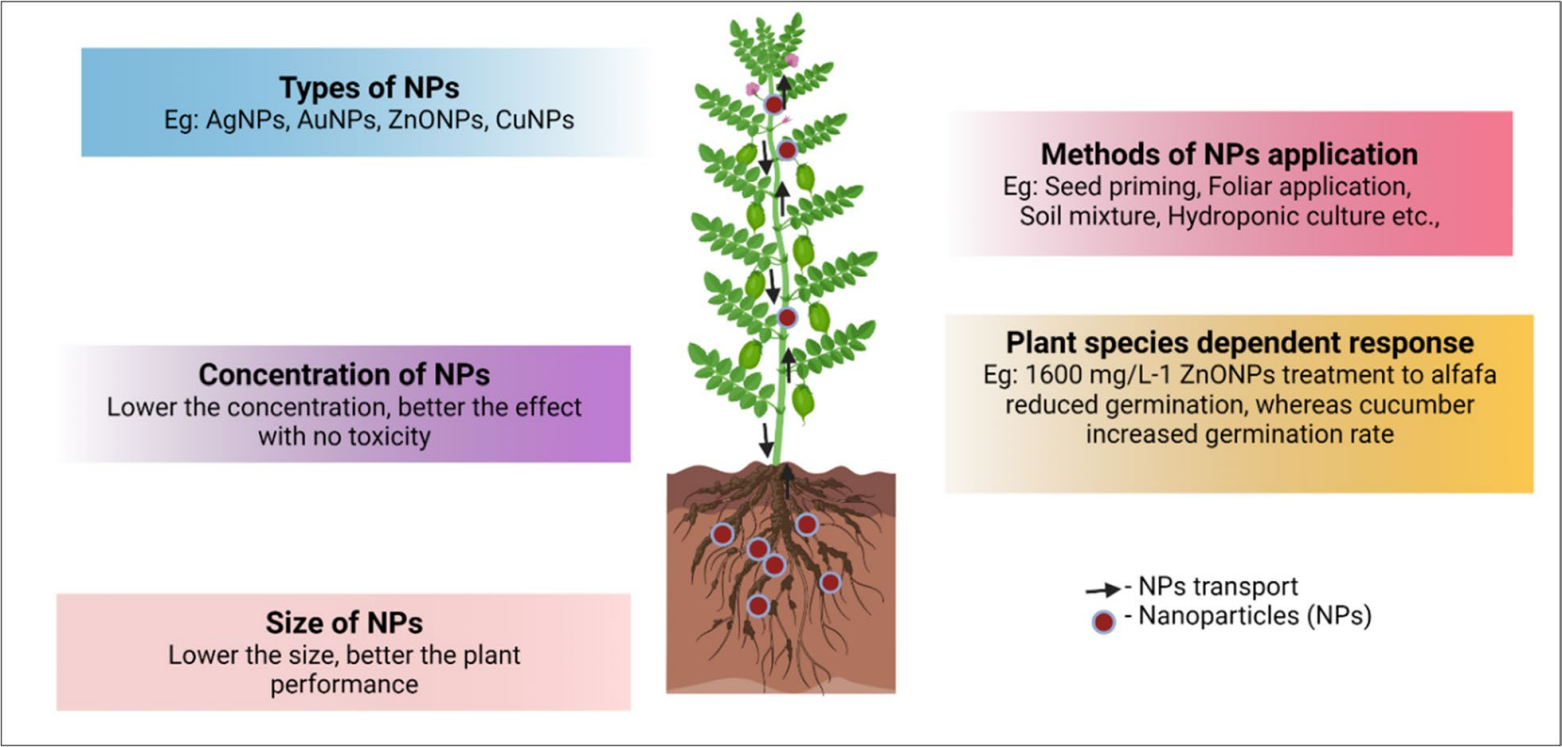
Factors to be considered during the treatment of nanoparticle application to plants. This figure was created using biorender software https://biorender.com/

The toxicity of nanoparticles (NPs) in plants is influenced by various factors, including the size and concentration of the NPs and the specific plant species involved (Fig. 3). Understanding these factors is crucial for assessing the potential risks associated with the use of NPs in agriculture.

#### Size

The size of NPs plays a significant role in their toxicity. Smaller NPs generally have a larger surface area, which increases their reactivity and potential for interaction with cellular components. This enhanced reactivity can lead to increased uptake and accumulation of NPs within plant tissues, potentially causing adverse effects.

#### Concentration

The concentration of NPs in the environment or applied to plants can also impact their toxicity. Higher concentrations of NPs may overwhelm the plant’s defense mechanisms and cellular detoxification processes, leading to cellular damage and stress responses.

#### Plant species

Different plant species exhibit varying sensitivities to NPs. Some plants may have mechanisms in place to tolerate or detoxify NPs more effectively, while others may be more susceptible to their adverse effects. The specific physiological and biochemical characteristics of each plant species can influence its ability to interact with and respond to NPs. It is important to note that the toxicity of NPs is not solely negative. NPs can also have beneficial effects on plant growth and development when used appropriately. Controlled application of NPs at lower concentrations and optimizing their size and surface properties can enhance nutrient uptake, improve stress tolerance, and promote overall plant health.

To better understand the toxicity of NPs in plants, ongoing research is focused on elucidating the underlying mechanisms of NP-plant interactions, studying the effects of different NP properties, and evaluating long-term impacts on plant growth, ecosystem dynamics, and food safety. Such research aims to ensure the safe and sustainable application of nanotechnology in agriculture while minimizing potential risks to plant health and the environment.

## Mode of metal nanoparticles application in plants

**Fig. 4:**
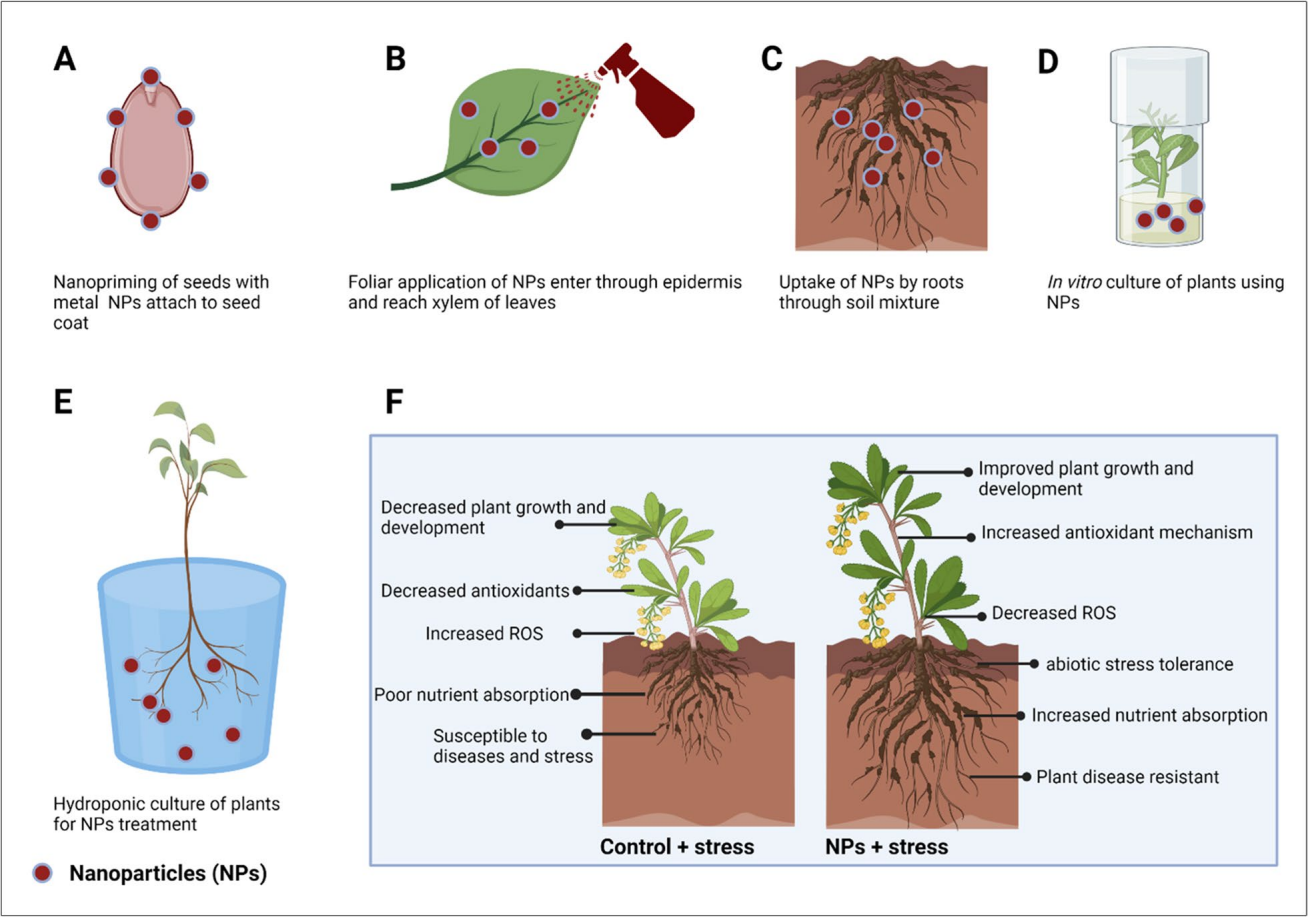
Different methods of NPs application and their physiological response in plants during stress and normal conditions. Based on the cited references, this figure was created using Biorender software https://biorender.com/

**Fig. 5:**
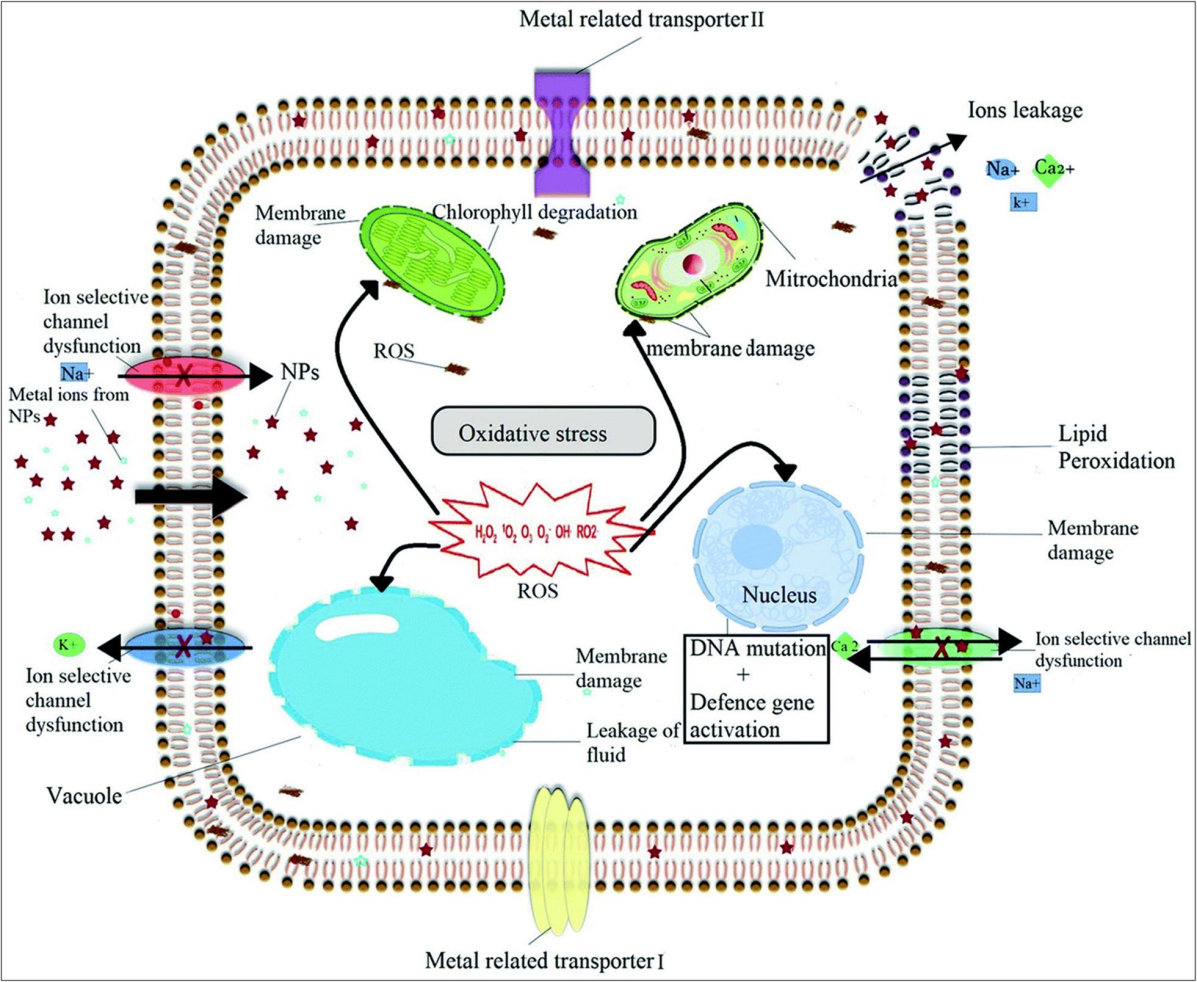
Diagram illustrating phytotoxicity of NPs through excessive ROS generation, damaging nuclear material, cell membranes, and organelles, ultimately resulting in cell death. This figure has been reprinted with permission from [35] Copyright, 2019

Nanoscience is a trending technology in plant science that uses various metal NPs as agrochemical carriers or fertilizers have been widely recognized for the past decade [26]. These NPs show action in seedling development, plant growth, germination, root growth [27], increased carbohydrate metabolism [28], ROS [29] transport of nutrients [30] during stressed and non-stressed conditions. For the application of NPs to plants, different methods of NPs treatment can be applied, including seed priming, foliar spray, and mixture with soil, hydroponic culture for sustainable agriculture (Fig. 4). However, one should cautiously choose treatment options for NPs application, as different treatment methods need different NPs concentrations, and the use of higher concentrations of NPs can cause negative effects on plants. It is also obvious that the wrong method of application can exhibit toxicity to plants, and therefore, securitizing the method depending upon plants, nanoparticles, and stress conditions are crucial for sustainable agriculture (Table 1). Figure 5 showing the possible phytoxicity response upon nanoparticle application in plants. Therefore, several aspects need to be considered before nanoparticle application to plants (Fig. 3). Among various methods of application, seed priming technology is the most popularly used method to induce the penetration of NPs through seeds via passive diffusion with water and this method has shown positive effects. During foliar application, stomatal permeation, epidermal absorption and internalization are the major ways to make foliage to absorb these nanoparticles. This has many advantages that include helping to fight plant diseases and pathogens, providing essential micronutrients through leaves that are rarely present in nutrition-deficient soil [31–34]. Unlike seed priming and foliar application, a soil mixture of NPs, hydroponic culture or in vitro application paves the way for the NPs to directly meet the ecosystem; and may cause negative impacts to soil ecosystem. Therefore, their use must be carefully considered. Owing to advantages and disadvantages of methods, seed industry is hunting for suitable priming agent and methods of application that could fit well for sustained agriculture and prevent detrimental effects on the ecosystem. Thus, in this section, we discuss about widely used metal NPs such as AgNPs, AuNPs, ZnONPs and CuNPs with respect to treatment options and their effect on physiological and biochemical responses of plants. In addition, we also elaborate the toxic or nontoxic effects of NPs based on the method of application, the concentration of NPs, and plant species.

**Table 1:** Mode of nanoparticle application influences phytotoxicity and their physiological function

Type of NPs	Conc. of NPs	Mode of NPs application	Physiological function	Phyto-toxicity	Condition	Reference
Silver nanoparticles (AgNPs)						
AgNPs	5 and 10 mg/L	Seed priming	Improved Water Intake, Seed Germination and starch Metabolism	Elevated ROS and H_2_O_2_	Normal	[28]
AgNPs	100 and 1000 mg/L	Seed priming	Decreased the germination and growth of rice seedlings	–	Normal	[36]
AgNPs	60 mg/L	Seed priming	Improved agro-morphological parameters, biochemical parameters, and enzymatic activities	–	Normal	[36]
AgNPs	150 mg/L	Soil	Increase of antioxidants, lipid peroxidation, and reduced contents of chlorophyll, carotenoids, total carbohydrate, and total soluble proteins	Accumulation of AgNPs in root > leaf > stem	Normal	[36]
AgNPs	200 mg/L	Vegetative growth stage	Increase of antioxidants, lipid peroxidation, and reduced contents of chlorophyll, carotenoids, total carbohydrate, and total soluble proteins	Accumulation of AgNPs in root > leaf > stem	Normal	[37]
AgNPs	10 and 15 mg/L	Seed priming and foliar application	Higher germination rate, increased chlorophyll contents, increased stomatal conductance, and higher seedling masses	Reduced diseased condition in seeds	Thermal stress	[38]
Gold nanoparticles (AuNPs)						
AuNPs	500–1000 µM and 5–11 mg/L	Soil	Increased the seed germination and vegetative growth	–	Normal	[44]
AuNPs	5–11 mg/L	Soil	Increased seed germination rate	–	Normal	[44]
AuNPs	5 to 15 ppm	Seed priming	Enhanced germination of naturally aged seeds, improved overall growth	–	Normal	[38]
AuNPs	10, 25, 50 and 100 mg/L	Foliar spray	10 ppm increased the number of leaves per plant and seed yield and 25 ppm increased total sugar content	–	Normal	[45]
AuNPs	20 µg/mL	Seed priming	Defense mechanism by improving plant growth and photosynthesis	–	Cold stress	[45]
AuNPs	0.1–10 mg/L	Size dependent (15, 30 and 40 nm)	Increase in chromosomal aberrations and decrease in mitotic index	–	Normal	[47]
AuNPs	10 mg/L	Size dependent (10 nm)	Decreased biomass and root length	–	Normal	[48]
AuNPs	22–25 nm	Hydroponic or soil mixing methods	-	Dose-dependent DNA damage	Normal	[49]
AuNPs	3.5 nm	Spherical-shaped AuNPs	Transporting in size-dependent mechanisms and translocating to cells and tissues	Exhibited leaf necrosis	Normal	[50]
Zinc nanoparticles (ZnONPs)						
ZnONPs	500 mg/mL	Soil	Improving the growth, chlorophyll contents, Zn contents	Reducing oxidative stress and cadmium (Cd) contents	Cd stress	[51]
ZnONPs	200 mg/mL	Foliar spray	Improving the growth, chlorophyll contents, Zn contents	Reducing oxidative stress and cadmium (Cd) contents	Cd stress	[51]
ZnONPs	1600 mg/L	Seed priming	Alfalfa was reduced to 40%, and tomato seeds by 20%, but increased cucumber seed germination	–	Normal	[53]
ZnONPs	100 mg/L	Seed	Increased germination rate	–	Normal	[54]
ZnONPs	1000 mg/L	Foliar application	Positive effect on plant height, stem diameter, chlorophyll content, fruit yield and biomass production	–	Normal	[51, 55–57]
ZnONPs	2000 mg/L	Foliar application	Negative effect on plant height, stem diameter, chlorophyll content, fruit yield and biomass production	Increase antioxidant activity	Normal	[51, 55–57]
ZnONPs	60 mg/L	Seed Priming	Maintain redox homeostasis by decreasing ROS generation; Increase antioxidant enzyme activities (SOD, peroxidase) and Low levels of Zn cannot elevate ROS due to poor activation of antioxidant machinery under stress conditions	Preventing cells from ROS attack under salt stress conditions	Salt stress	[58, 59]
ZnONPs	90 mg/L	Soil	Triggered localization of ZnONPs in vacuoles and chloroplasts; Reversed abnormal modifications to chloroplast, mitochondria, and cell wall	Stimulated antioxidant enzymes, enhanced osmolyte contents; No phytotoxicity observed under heat stress for alfalfa plants	Heat stress	[58, 59]
ZnONPs	100 mg/L	Foliar application	Improved drought-associated detrimental effects and growth-promoting effect	–	Normal and drought	[58, 59]
ZnONPs	400 mg/L	Foliar application	Increased oxidative stress	–	Normal	[60]
ZnONPs	400 mg/L	Foliar Application with Silicon Modification	Improved stability, hydrophilicity, and salt tolerance	–	Normal	[60]
ZnONPs	500 mg/L	Soil mixture	Increased Zn in roots; Root elongation; Translocation of Zn to aerial parts	Increased H_2_O_2_ accumulation in leaves; Reduced antioxidant enzymes (CAT, APX)	Normal	[61]
Copper nanoparticles (CuNPs)						
CuNPs	4.44 mg/L	seed priming	Improved plant biomass in normal and drought conditions	–	Normal and drought	[61]
CuNPs	250 mg/L	Seed priming	Increased bioactive components (vitamin C, lycopene, total phenols, flavonoids), antioxidant enzyme accumulation (CAT, SOD)	–	Normal and drought	[62]
Cu	0–20 mg/L	Hydroponic Culture	Reduced root length in lettuce and alfalfa; Translocation of nCu observed in dose-dependent manner	Alfalfa more sensitive to nCu compared to lettuce	Normal	[71]
nCu	10 and 20 mg/L	Hydroponic Culture	Reduced water content, root length, dry biomass; Modified defense-related metabolites	–	Normal	[6, 70]
Cu@CuO and nCuSO_4_.5H_2_O	10 and 20 mg/L	Hydroponic Culture	Reduced water content, root length, dry biomass; Modified defense-related metabolites	–	Normal	[6, 70]
nCu(OH)_2_-b	1050 mg/L to 2100 mg/L	Foliar spray	Increased leaf biomass; Changes in metabolites indicating defensive response	–	Normal	[71]
nCu/Kg	200–800 mg/mL	Soil	Increased Cu accumulation in roots; Detrimental effects in stem, leaves, and fruits	–	Normal	[71]
nCu(OH)_2_-b	10 mg/L	Soil	Arrested photosynthesis, stunted growth in Clarika unguiculata	High light levels and limited soil conditions	Normal	[71]

### AgNPs

AgNPs are a commercialized nanomaterial used in the medical field as antimicrobial agents and personal care products. Due to their eco-friendly properties, recently, adequate interest has been developed among plant biologists to use AgNPs as an efficient nanomaterial in the agricultural sector to improve seed germination, plant growth and development under environmental stress conditions. Majorly, AgNPs are applied to plants by seed priming technology, foliar application or through soil mixture methods. Also, the improvement in plant performance were highly modulated by the type of application used. Therefore, in this section, we will understand the major differences in plant growth and performance during normal and stress conditions when different methods of AgNPs treatments are adapted. A biocompatible AgNPs were synthesized using kaffir lime leaves extract to evaluate their ability to improve rice seed germination and starch metabolism after seed priming application using 5 and 10 mg/L AgNPs under normal conditions [28]. AgNPs penetrated the seed coat and improved the water intake, elevated ROS and H_2_O_2_, and improved seed germination as well as starch metabolism compared to silver nitrate (AgNO_3_) treatment. These observations aided the hypothesis of nanopriming of seeds with AgNPs involving the loosening the cell wall of seed coat and endosperms at low concentrations [28]. Moreover, the minimal nanoparticle concentration utilized in seed priming not only reduces production costs but also mitigates the widespread dispersion of nanoparticles in the environment. As a result, seed priming emerges as an eco-friendly approach.

**Fig. 6:**
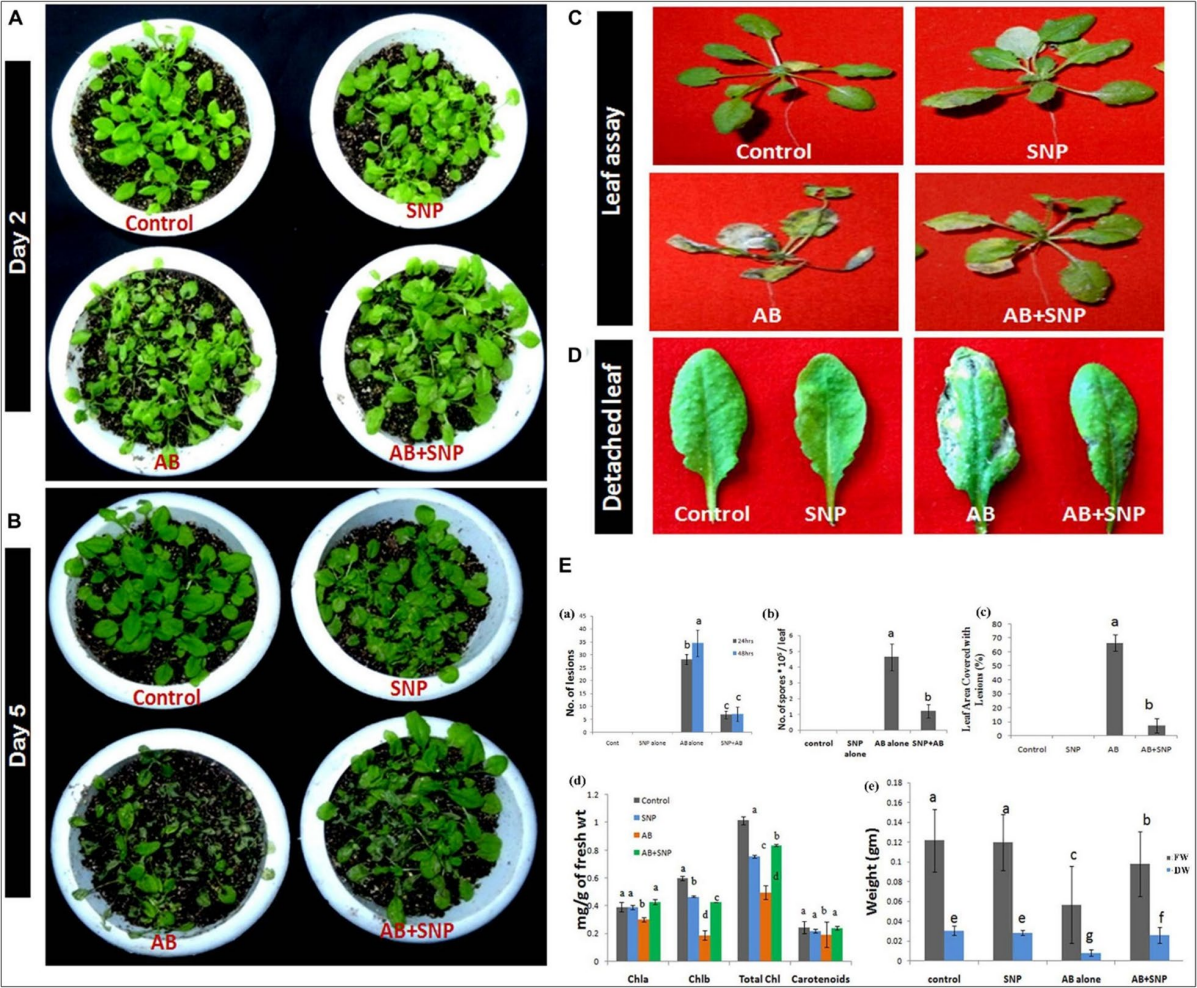
Photograph showing effect of silver nanoparticles in reducing disease severity after **(A)** 48 h (day 2) post infection **(B)** Day 5 post infection **(C)** reduction in necrosis of leaves **(D)** reduction in number of lesions formed per leaf **(E)** Assessment of disease parameters in terms of **(a)** number of lesions **(b)** number of spores **(c)** leaf area covered with lesion **(d)** chlorophyll content **(e)** fresh and dry weight in silver nanoparticles pre-treated plants as compared to other treatments. Cont-Control, SNP-Biogenic silver nanoparticles lone, AB-*A. brassicicola* infected plants, AB+SNP-*A. brassicicola* infected, treated with SNP, FW-fresh weight, DW-Dry weight. Values are the means ± SD of three replicates. Means sharing different alphabets “a”, “b” differ significantly from each other at *p* ≤ 0.05. This figure has been reprinted with permission from [40] Copyright, 2020

The application of seed priming also minimizes the dispersal of the larger number of NPs into ecosystems as NPs treatment applied to seeds did not reach soil and therefore, can be suggested as a promising technique for its commercial use. However, one should be cautious when using seed priming of AgNPs to improve seed germination and growth of rice plants due to their size and concentration-dependent responses. Rice seeds soaked with different sizes (20, 30–60, 70–120 nm) and concentrations of AgNPs (100 and 1000 mg/L) decreased the germination and growth of rice seedlings. Therefore, it is crucial to consider optimum sizes and concentrations of AgNPs to prevent their phytotoxic effects in rice seedlings. During seed priming of 60 mg/L AgNPs improved agro-morphological parameters, biochemical parameters, and enzymatic activities in sunflower plants. Whereas through combined methods i.e., seed priming and foliar application, improved plant yield, seed quality and secondary metabolite contents of the sunflower plants, indicating that each method of application can be recruited to improve unique characteristics of sunflower plants. On the other hand, 150 mg/L AgNPs through soil application increased the toxicity in sunflower plants by the accumulation of AgNPs in root > leaf > stem, which was reflected from the increase of antioxidants, lipid peroxidation, and reduced contents of chlorophyll, carotenoids, total carbohydrate, and total soluble proteins [36]. Similar phytotoxic effects were reported at the vegetative growth stage compared to that of germination in both cucumber and wheat plants that were exposed to 200 mg/L of AgNPs through in vitro application [37]. In another study, positive effect of urea with low concentrations of AgNPs (10 and 15 mg/L) through the application of seed priming and foliar application has been showed in terms of reduced diseased condition in seeds, higher germination rate, increased chlorophyll contents, increased stomatal conductance, and higher seedling masses in oilseed rape and cucumber under thermal stress [38]. In eggplant, foliar spray of AgNPs under drought conditions improved growth parameters, photosynthetic pigments, proline, hydrogen peroxide (H_2_O_2_) and antioxidant activities [39], suggesting the use of AgNPs can replace harmful pesticides and highly concentrated mineral fertilizers. The pre-treatment of biosynthesized AgNPs with *A. brassicicola* showed significant reduction in lesions compared to *A. brassicicola* alone treated plants (Fig. 6**).** These results suggest that the appropriate selection of AgNPs application can positively regulate the plant growth and performance under stress and non-stressed conditions.

### AuNPs

**Fig. 7:**
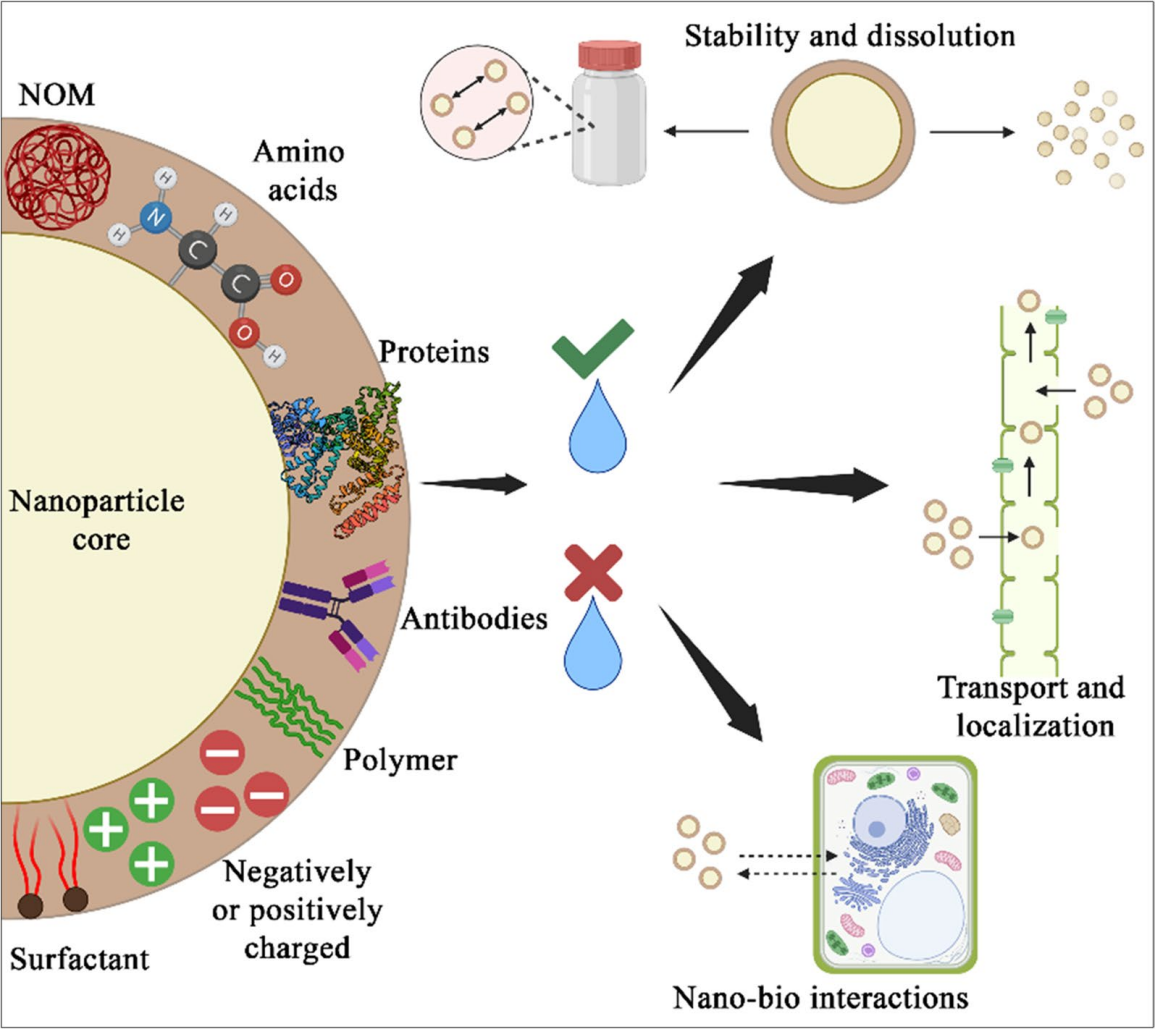
Various types of coatings on the nanoparticles and their impact in the agriculture field: The metal nanoparticles can be coated with natural organic matter (NOM), amino acids, proteins, antibodies, polymers, surfactants, and negatively or positively charged moieties. Such coatings make the surface of nanoparticles either hydrophilic or hydrophobic and govern their stability in aqueous suspension, dissolution, transport in plants, and interactions with plant cells

AuNPs were widely used in various fields, including medicine, biology, chemistry, physics, electronics, cosmetics, and so on. However, there is only a minimal number of studies reported in plants concerning plant growth, development and phytotoxicity. Generally, the plants exposed to AuNPs exhibited both positive and negative effects, which are majorly dependent on concentration, particle size, shape, and species [41]. The method of NP application to plants is also crucial, i.e., whether the uptake is through leaves, roots, or seeds [42]. In vitro study in Arabidopsis seedlings indicated that direct treatment of the smallest AuNPs of 10 nm at the lowest concentration induced root hairs but decreased the number and length of lateral roots with higher particle concentrations of AuNPs [43]. In glory lilly, 25 nm sized AuNPs at 500–1000 µM concentrations treated to soil for 40 days increased the seed germination and vegetative growth [44]. Similarly, in maize, 11 nm AuNPs treated at a concentration of 5–11 mg/L to soil for 10 days increased seed germination rate. Another study on AuNPs synthesized using ecofriendly rhizome extract of galanga plant when applied through seed priming method, enhanced germination of naturally aged seeds of maize plants and improved overall growth, without exhibiting toxicity at 5 to 15 ppm concentration [38]. A foliar spray application of 10, 25, 50 and 100 mg/L AuNPs were applied to *Brassica juncea*, showed that 10 ppm AuNPs increased a number of leaves per plant and seed yield. However, total sugar content increased when 25 ppm AuNPs were applied, indicating that the lower concentration of AuNPs were sufficient to enhance physiological and biochemical parameters of *Brassica* sp.[45]. AuNPs are capable of inducing stress-related mechanisms to provide resistance to stress conditions in plants. For example, a foliar application of biosynthesized AuNPs reduced salt stress by maintaining correct ratio of reactive oxygen species to reactive nitrogen species (ROS/RNS ratio) and improving defense mechanism in wheat seedlings. Thus, such AuNPs can be used as an alternative for chemical fertilizers to maintain nutritive status, prevent post-agricultural losses, and mitigate abiotic stresses [46]. In wheat, using seed priming method, 20 µg/mL AuNPs acted as a signaling molecule under cold stress and activated a defense mechanism by improving plant growth and photosynthesis [47]. Overall, AuNPs work best at lower concentrations to improve physiological parameters under normal and abiotic stress conditions (Fig. 7). In addition to a physiological response, very few studies on the toxicity of AuNPs in plants were also reported. AuNPs treatment affected the growth and development of various plants and showed contradictory effects depending on the mode of NP application. When onion plants were treated with 15, 30 and 40 nm sized AuNPs in vitro for 4 h at the concentration of 0.1–10 mg/L, authors observed increase in chromosomal aberrations and decrease in mitotic index [48]. In barley, 10 nm sized 10 mg/L AuNPs treated in hydroponic medium for 2 weeks decreased biomass and root length [49]. The administration of spherical gold nanoparticles (AuNPs) through hydroponic or soil mixing methods demonstrated toxic effects. Specifically, in the case of tobacco plants, the application of 22–25 nm AuNPs in increasing concentrations resulted in dose-dependent DNA damage. [50]. Similarly, spherical-shaped AuNPs sized 3.5 nm exhibited leaf necrosis effect after 14 days of exposure by transporting in size-dependent mechanisms and translocating to cells and tissues resulting in phytotoxicity [51]. To conclude, AuNPs exhibited toxicity in vitro, hydroponic and soil treatment irrespective of plants used and therefore, seed priming can be adapted for the treatment with AuNPs to improve plant immunity without exhibiting toxic effects.

### ZnONPs

Zinc (Zn) is a crucial element for the plant growth and development because carbohydrate, protein, and chlorophyll formation significantly decrease in plants that devoid of Zn. The use of zinc oxide (ZnO) or ZnSO4.7H_2_O) as fertilizers are limited due to their low solubility in soil and poor bioavailability of zinc to plants. Therefore, ZnONPs have got special attention in agriculture field. Zinc nanoparticles have shown to possess the ability to penetrate the seed coat that resulted in increased aquaporin genes involved in water uptake, seed vigor, bioavailability, solubility in soil, slow and gradual release. The ZnONPs primed seeds had increased influence on growth and physiology status compared to bulk ZnSO_4_ treatment, perhaps due to greater ability to be absorbed and assimilated due to nano size. In a study when Zn is added to the primed solution, it improved budding and seedling growth of wheat seedlings, probably because Zn is involved in the early stages of coleoptile and radicale development [52]. The increase of α-amylase in ZnONPs treated seeds can increase availability of soluble sugars which in turn increase the germination rate, seedling length, seed water uptake for improving overall metabolic activity [47]. Compared to seed priming, foliar spray of ZnONPs used higher amount of ZnONPs but their use is quite low compared to soil mixture strategy in wheat plants. For instance, soil application of ZnONPs to wheat tissues used total amount of 500 mg for four plant replicates [53], whereas ZnONPs using foliar spray to wheat tissues used only 200 mg for four replicates denoting the efficient use of ZnONPs for bio-fortification in plants. This study also showed the effectiveness of ZnONPs in improving the growth, chlorophyll contents, Zn contents, and by reducing oxidative stress and cadmium (Cd) contents under Cd stressed water-deficient wheat plants. We also found that seed priming of ZnONPs on germination generally depends on the concentration of NPs used, and choice of plant species [54]. For instance, under 1600 mg/L ZnONPs treatment, germination rate of alfalfa was reduced to 40%, and tomato seeds by 20% but increased cucumber seed germination compared to control, indicating that higher concentration of ZnONPs affect the quality of germination. In the egg plant seeds treated with 100 mg/L ZnONPs, the germination rate was increased via reducing the seed dormancy [51, 55–57] compared to foliar spray and soil mixture. Similarly, there were concentration-based physiological responses observed in habanero pepper plants when foliar application was applied. The foliar spray with different concentrations of ZnONPs showed different functionalities. 1000 mg/L of ZnONPs foliar spray on pepper plants caused positive effect on plant height, stem diameter, chlorophyll content, fruit yield and biomass production; but 2000 mg/L of ZnONPs foliar treatment negatively affected the above parameters. Same dose resulted in increase in fruit quality, capsaicin content, dihydrocapsaicin, total phenols and flavonoids in fruits, and increase antioxidant activity suggesting that concentration-dependent ZnONPs effects in pepper plants. Additionally, the seed priming and foliar application of ZnONPs has also shown to exhibit abiotic stress tolerance. The primed wheat seeds used ZnONPs (60 mg/L) to maintain redox homeostasis by decreased ROS generation, and increased antioxidant enzyme activities such as superoxide dismutase (SOD), peroxidase, thus preventing cells from ROS attack under salt stress conditions. It is well known that low level of Zn is unable to elevate ROS due to poor activation of antioxidant machinery under stress conditions [58, 59]. Another study showed the application of 90 mg/L ZnONPs prior to heat stress to alfalfa plants triggered localization of ZnONPs in vacuoles and chloroplasts, reversed the abnormal modifications to chloroplast, mitochondria and cell wall by stimulating antioxidant enzymes and enhancing osmolyte contents, whereas in soil application that did not happen [60]. When cucumber is treated with 100 mg/L ZnONPs through foliar application, the nanoparticles improved drought-associated detrimental effects by regulating morphological, physiological, and biochemical attributes. Similar studies of foliar application of ZnONPs in improving growth-promoting effect have been reported in wheat, cucumber, and eggplants under normal and drought conditions indicating the role of ZnONPs as a promising fertilizer to improve growth and stress conditions.

As far as toxicity is concerned, the foliar application of ZnONPs showed increased oxidative stress at 400 mg/L. Whereas, the surface modification of 400 mg/L ZnONPs with silicon (Si) improved the stability, hydrophilicity of ZnONPs with improved salt tolerance effect with no phytotoxicity. Thus suggesting the use of ZnO-SiNPs compared to ZnONPs in pea plants [61]. The negative effect was also observed when ZnONPs at concentration of 500 mg/L was mixed in soil. This mixing increased Zn in roots compared to bulk Zn treated pea plants leading to root elongation, translocation of Zn to aerial parts, and increased H_2_O_2_ accumulation in leaves with the reduction in antioxidant enzymes such as catalase (CAT), and ascorbate peroxidase (APX). Specifically, after twenty-five days of treatment, there was a significant reduction in chlorophyll content, and increase in lipid peroxidation, indicating the highest toxicity due to accumulation in ZnONPs treated pea plants that can cause huge negative impact on ecology and food chain [62]. Therefore, it is necessary to select the NP treatment options carefully when considering crop health improvement using nanomaterials. Similarly, in chickpea plants devoid of Zn showed an increased in malondialdehyde (MDA) thus resulting in decreased biomembrane integrity. However, ZnONPs primed seeds reversed Zn content and decreased MDA by protecting membrane integrity [63]. Overall, ZnONPs applied through seed priming showed a positive effect without exhibiting toxicity to plants compared to soil mixture and foliar spray. Functionalization of ZnONPs can be recommended to minimize the toxic effects of ZnONPs when the foliar spray is used. In summary, choosing the optimum concentration of NPs related to the application method is crucial for getting benefits out of NPs.

### CuNPs

**Fig. 8:**
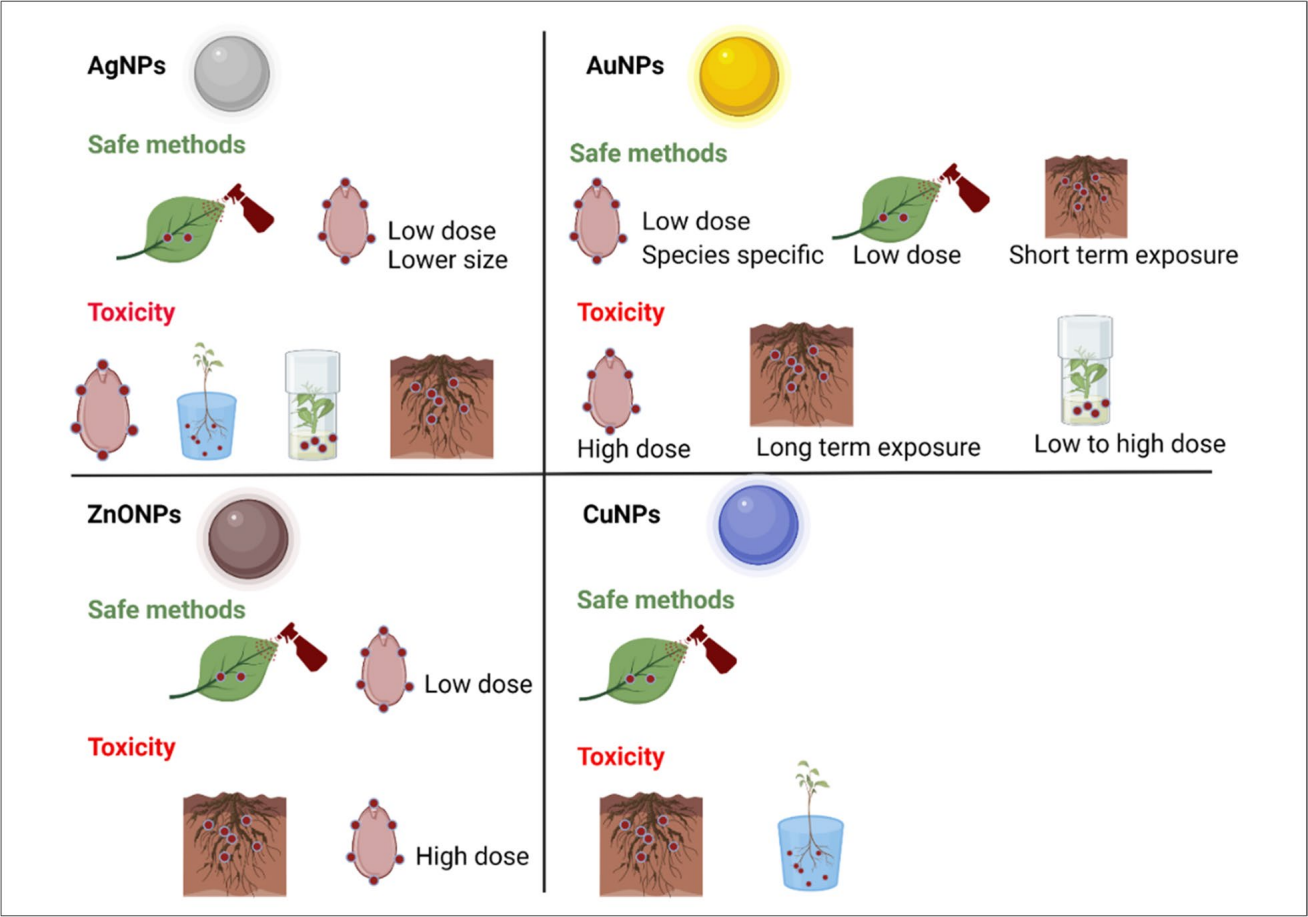
Constructive and destructive effects of different methods of NPs application in plants depending on this review. This figure was created using biorender software https://biorender.com/

Copper (Cu) is another micronutrient for plant growth and development that is involved in many biochemical reactions of plant cells. There are also several studies on the application of CuNPs to improve seed yield and quality under normal and stress conditions. The dose of Cu in the nano or microform (nCu, nCuO, nCu (OH)_2_-a, nCu (OH)_2_-b, µCu and µCuO) and CuCl_2_ are crucial for showing beneficial or detrimental effects in the terrestrial ecosystem. For instance, cilantro plants treated with nCuO from germination to harvesting time has more negative effects on germination, chlorophyll content, and plant biomass compared to all other Cu based particles indicating the role of nCuO role in exhibiting negative nutritive effects on cilantro plants [4]. Similarly, studies showed that seedlings such as Syrian barley[64], soybeans and chickpeas [65], mung beans and wheat [66], radish [6, 67, 68], lettuce [6, 69] were affected at 0.5 mM nCuO, 500 mg/L, 335 and 570 mg/L, 10 mg/L, 0.1 mg/L inhibited growth rate. Surprisingly, the method of CuNPs application plays vital role in determining the nanoparticle toxicity of plants. For instance, seed priming with 4.44 mg/L CuNPs positively improved plant biomass in normal and drought conditions. Whereas for improving the quality of tomato fruits, 250 mg/L CuNPs are recommended as it increase bioactive components such as vitamin C, lycopene, total phenols, and flavonoids by increased accumulation of antioxidant enzymes such as catalase (CAT) and superoxide dismutase (SOD) [6, 70]. This indicates that the differences in concentration of CuNPs and its impact on plants also fluctuate depending on the stress conditions. Plants such as lettuce and alfalfa grown in hydroponic culture with nCu, nCuO, nCu(OH)_2_-a, nCu(OH)_2_-b, µCu, and µCuO from 0 to 20 mg/L Cu concentration showed reduced root length in both the plants. Specifically, the translocation of nCu to leaves in lettuce plants was observed only after treatment with 10 and 20 mg/L concentrations and specifically, in alfalfa plants the translocation of nCu was observed in the dose dependent manner. Thus, proving that the alfalfa was more sensitive to nCu compared to lettuce plants. Overall, nCu treatments produces differential responses even between the plants of dicots. Similarly, lettuce grown using hydroponic culture with 10 and 20 mg/L Cu@CuO and nCuSO_4_.5H_2_O showed reduced water content, root length, dry biomass and modified defense-related metabolites in roots [71]. However, foliar spray of 1050 mg/L to 2100 mg/L nCu(OH)_2_-b for the last four weeks before harvest did not exhibit negative effects instead, it increased leaf biomass. However, there was significant changes in the metabolite such as cis-caffeic acid, chlorogenic acid, 3,4-dihydroxycinnamic acid, dehydroascorbic acid occurred, demonstrating the occurrence of defensive response against oxidative stress. Additionally, when cucumber plants exposed to 200–800 mg nCu/Kg in soil increased Cu accumulation in roots and able to translocate significantly to stem, leaves and fruits, causing detrimental effects. Similarly, when *Clarika unguiculata* (mountain garland) were exposed to 10 mg/L nCu(OH)_2_-b in soil, it completely arrested photosynthesis and caused stunted growth in high light levels and limited soil conditions [72]. This indicated that seed priming and foliar spray of CuNPs are comparatively better than hydroponic culture and soil treatment for improving the quality of dicot plants (Fig. 8). There are also studies reported on CuNPs against biotic stress tolerance.

Micronutrients applied in the form of nanoparticles showed efficacy in increasing vegetable and crops yield and decreasing fusarium diseases. A foliar application of 500 μg/mL CuONPs to chrysanthemum plant with or without *Fusarium oxysporum f. sp. chrysanthemi* reduced disease severity with an increase in dry biomass, plant height, horticulture quality [73]. Thus, indicating that CuONPs able to improve overall performance of chrystanemum plants under stress or non-stress condition. Additionally, priming of maize seeds with CuNPs exhibited drought tolerance by maintaining leaf water status, chlorophyll, and carotenoids with decreased ROS and increased antioxidant enzymes such as SOD, ascorbate peroxidase (APX), and anthocyanin contents [74].

## Impact of metal nanoparticles coating in agriculture

The NPs are continuously explored for their application in agriculture with the aim of achieving sustainable agriculture, delivery of nutrients or pesticides to crops, improvement in crop yield and agriculture performance as well as reducing the waste, and treating the infectious diseases in plants. NPs having a size below 100 nm show many advantages, such as increased internalization from plant parts (e.g., leaves, roots, etc.), high reactivity owing to the increased surface area to volume ratio, and greater bioavailability in plants. But these NPs suffer from drawbacks of agglomeration, instability, dissolution in aqueous suspension, and soil adsorption, which could lead to their decreased activity, poor transport in the plant, phytotoxicity, and toxicity to the ecosystem associated with the plant. In order to improve the effectiveness of these NPs, they can be coated with various chemical moieties, which help in reducing agglomeration and regulating dissolution. Such chemical moieties include natural organic matter (NOM), polymers, zwitter ionic surfactants, proteins, etc. Furthermore, these coatings also help in translocation of NPs inside the plants as well as they actively participate in nano-bio interactions upon entry into the cell. To put these coatings on NPs, various methods like physical adsorption, chemical adsorption, covalent linkage, association via hydrogen bonding, electrostatic interactions or hydrophobic interactions has been used [6]. Humic acids or humic substances, a major component of the organic fraction of soil have been used widely to coat the NPs that are meant for agricultural applications [75, 76]. Yoon et al. have shown that hydroxyapatite NPs coated with humic substances help in the synergistic release of nutrients and stimulants to crops, which can be potentially used as crop fertilizer. Authors showed that the humic substances offered excellent stability to hydroxyapatite NPs and prevented their sedimentation in an aqueous medium. Authors attributed this property to the electrostatic repulsion between negatively charged humic substances on the surface of NPs as well as to the hydrophilicity of multiple oxygen-based functional groups of humic substances. To prove the agronomical effectiveness of humic substances coated nanoparticles, authors showed that the height, fresh and dry weight of maize plants was increased significantly after NPs treatment. Additionally, the rhizosphere microbial community analysis showed that plant rhizosphere treated with humic substances coated NPs showed an increase in the microbiome associated with symbiotic plant–microbe interactions. Thus, the synergistic action came from fast dissolving hydroxyapatite nanoparticles (that provided calcium and phosphorous) and multiple beneficial effects of humic substances on plant and plant microbiome [77]. Baile Xu et al. showed another use of humic substances for sorption of common soil contaminants that usually reduces the usefulness of soil for agriculture if not removed. The authors showed that the sorption of hydrophobic organic contaminants like pentachlorophenol and phenanthrene could be improved using humic acid-coated Fe_3_O_4_NPs. They attributed hydrogen-bonding interactions, hydrophobic interactions, and π-π interactions between humic acid and organic contaminants to the sorption capacity of humic acid-coated hematite NPs [78]. Ian L. Gunsolus et al. have also reported similar findings in the case of AgNPs. The authors observed that owing to the coating of fulvic acid and humic acid-based NOM, the AgNPs gain exceptional stability in the natural aquatic environment. Such coating prevents the aggregation of nanoparticles and their dissolution and conversion into Ag ions, which ultimately leads to the availability of NPs in their intact form for further action. However, the authors did not evaluate the effect of NPs in specific environments (e.g., agricultural soil, natural water reservoir etc.) [79]. In another study, Peiguang et al. demonstrated the effect of surfactant coating on foliar delivery of nanoparticles. Authors proved that the surface charge, as well as the size of NPs govern the delivery of carbon dots (C-dots), cerium oxide (CeNPs), and silica (SiO_2_NPs). They showed that triton X-100 and silvet L-77 improved the delivery of NPs in cotton and maize leaves. Depending on size and charge on the nanoparticle surface, these nanoparticles accumulated in various parts of leaves starting from extracellular spaces to cell organelles. Such translocation and accumulation were found to be higher in the case of amine-rich positively charged coating of nanoparticles as compared to negatively charged nanoparticles [80]. Although the authors did not comment on the type of interactions between nanoparticles and leaf cells, concerning their results, one can safely assume that electrostatic interactions play the dominant role in nanoparticle translocation with little contribution from hydrophobic interactions owing to the presence of surfactant on the nanoparticle surface. Avellan et al. have made similar observations in the case of AuNPs. They showed that foliar uptake of nanoparticles, their transport from leaves to rhizosphere, and their translocation in wheat were directly dependent on the coating of AuNPs. Contrary to what showed by Peiguang et al. Avellan et al. stated that irrespective of zeta potential and surface charge on nanoparticles, polyvinylpyrrolidone (PVP) coated AuNPs showed significantly higher uptake as compared to citrate coated AuNPs. Authors attributed this uptake solely to the hydrophobic interactions and not to the electrostatic interactions. However, for same nanoparticles authors reported impairment in photosynthesis in wheat. Increased hydrophobicity of nanoparticles due to presence of PVP, increased hydrophobic interactions with plant cell membrane, increased uptake, and entry into mesophyll of wheat plant leaves were main reasons for reduced photosynthesis [81]. In same line of research, Yiming Su et al., tested effect of various coatings (PVP, Gum Arabic and citrate) on translocation of AgNPs in citrus tree. They observed that PVP and gum arabic coated NPs did not show aggregation. Furthermore, among steric, osmotic, elastic repulsive interactions, the NPs predominantly showed steric repulsive interactions with xylem and phloem cell wall enabling their mobility inside plant part. Depending on route of administration (foliar application, branch feeding, soil drenching, and tree trunk injection), the nanoparticles achieved higher concentration within short duration owing to their increased mobility because of various coatings. Authors have suggested that due to improved delivery to various parts of citrus plant, these nanoparticles can be used as gene delivery vehicle and/or antimicrobial agents in agriculture field [82]. Manli Yu et al. developed pesticidal polymeric NPs by in situ loading of abamectin onto carboxyl/acetyl/amine grafted polylactic acid NPs. The authors predicted that various functional groups on the surface of NPs interact with glycoside moieties, fatty alcohols, fatty acids, and fatty aldehydes on the surface of cucumber leaves. They stated that for acetyl-functionalized NPs, hydrogen-bonding interactions were highly predominant. In case of negatively charged carboxyl functionalized NPs, hydrogen-bonding interaction were present but due to electrostatic repulsive interactions exhibited at the same time, the effect of hydrogen-bonding interactions was weak. Conversely, for positively charged amine-functionalized NPs, electrostatic attraction and Schiff's base associated covalent linking between fatty acids and fatty aldehydes along with hydrogen-bonding interactions rendered them strongly interacting NPs. Therefore, the deposition and retention of these pesticide NPs were observed in the order of amine-functionalized NPs > acetyl functionalized NPs > carboxyl functionalized NPs [83]. For titanium dioxide nanoparticles (TiO_2_NPs), the effect of positively charged hydrophobic (dimethicone) and negatively charged hydrophilic (glycerol) coating has been evaluated in basil plants. Owing to electrostatic attraction and hydrophobic interactions, dimethicone coated TiO_2_NPs showed higher accumulation in basil roots. Upon treatment, both hydrophilic as well as hydrophobic nanoparticles, significantly affected nutrient accumulation in basil roots and shoots. Although there was no significant change in chlorophyll contents, the hydrophobic TiO_2_NPs reduced the biomass significantly. Both types of coatings on TiO_2_NPs had a negative impact on plant growth, suggesting the role of coating moiety in the response of the plant to the nanoparticle treatment [84]. Beneficial effects of various hydrophobic/hydrophilic moieties and that protein have been reported in soybean and fava bean plants, respectively. Majumdar et al. synthesized cadmium sulfide quantum dots (QDs) capped with trioctylphosphine oxide (TOPO), PVP, mercaptoacetic acid (MAA), and glycine (Gly). They reported that the NPs dissolution, entrapment, localization, and metabolic activity in plants are directly dependent on the coating of the QDs. With respect to stability, MAA-QDs were highly stable and released very low amount of cadmium ions. Owing to negative charge on their surface, the entrapment was very low and majority of QDs were loosely associated with outer parts of roots. This finally resulted in low translocation of QDs. On the contrary, TOPO-QDs were highly unstable and formed aggregates. Due to positive surface charge as well as hydrophobicity of TOPO-QDs, they showed good entrapment; however, owing to hydrophobic interactions with the lipids, they were immobilized in plant cell membrane leading to low translocation in plant shoots. PVP-QDs showed efficient and maximum translocation from roots to shoots because of positively charged surface with desirable stability in aqueous suspensions. In case of these three QDs, the metabolic activity of soybean plant was not significantly affected. But Gly-QDs showed more stability than TOPO-QDs and showed negative impact on metabolic activity of plant. Hence, authors have concluded that nanoparticles with particular coating not only affect their fate but may also show impact on plant health and food chain associated with it [76]. Spielman-Sun et al. prepared LM6-M antibody and bovine serum albumin (BSA) coated AuNPs for their targeted delivery to stomata of fava bean plant. LM6-M antibody shows specific affinity towards α-1,5-arabinan present in stomata of fava bean leaves. Thus, antibody coated AuNPs showed specific accumulation in stomata. On the other hand, BSA provided amphiphilic nature to nanoparticles leading to their accumulation in trichome hairs on the leaves. Although exact nature of interactions is not known for BSA coated AuNPs, authors predicted that the electronegativity of BSA and its amphiphilic nature plays the dominant role in their interactions with polar groups in trichomes [85].

## Delivery of biomolecules to plants

**Fig. 9:**
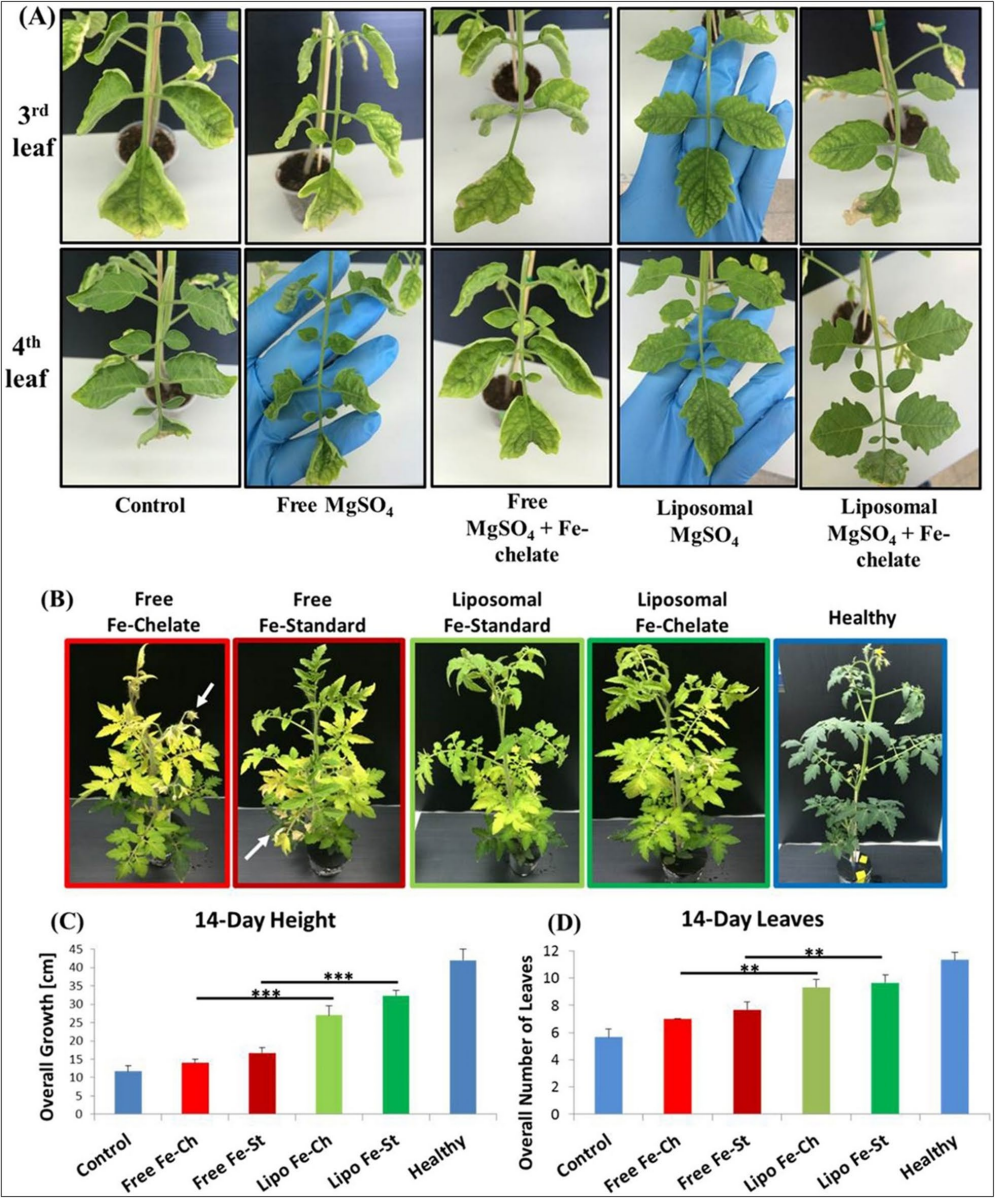
Nanotechnology was used to supplement Mg and Fe deficiencies in tomato plants. In the upper panel, a liposomal formulation was applied to tomato seedlings' apical leaflet, resulting in improved recovery compared to non-encapsulated formulations. In the lower panel, tomato plants grown hydroponically in Fe-deficient media were treated with liposomal Fe-chelate, showing moderate yellowing but promoting healthy new growth. On the other hand, non-liposomal Fe-chelate and free Fe-standard treatments led to severe yellowing and necrosis. Nanoparticle-encapsulated iron significantly improved growth patterns, outperforming non-encapsulated treatments. Statistical analysis confirmed the significant impact of the nanoparticle-based approach (*p < 0.05, ***p < 0.001).This figure has been reprinted with permission from [90] Copyright, 2018

**Fig. 10:**
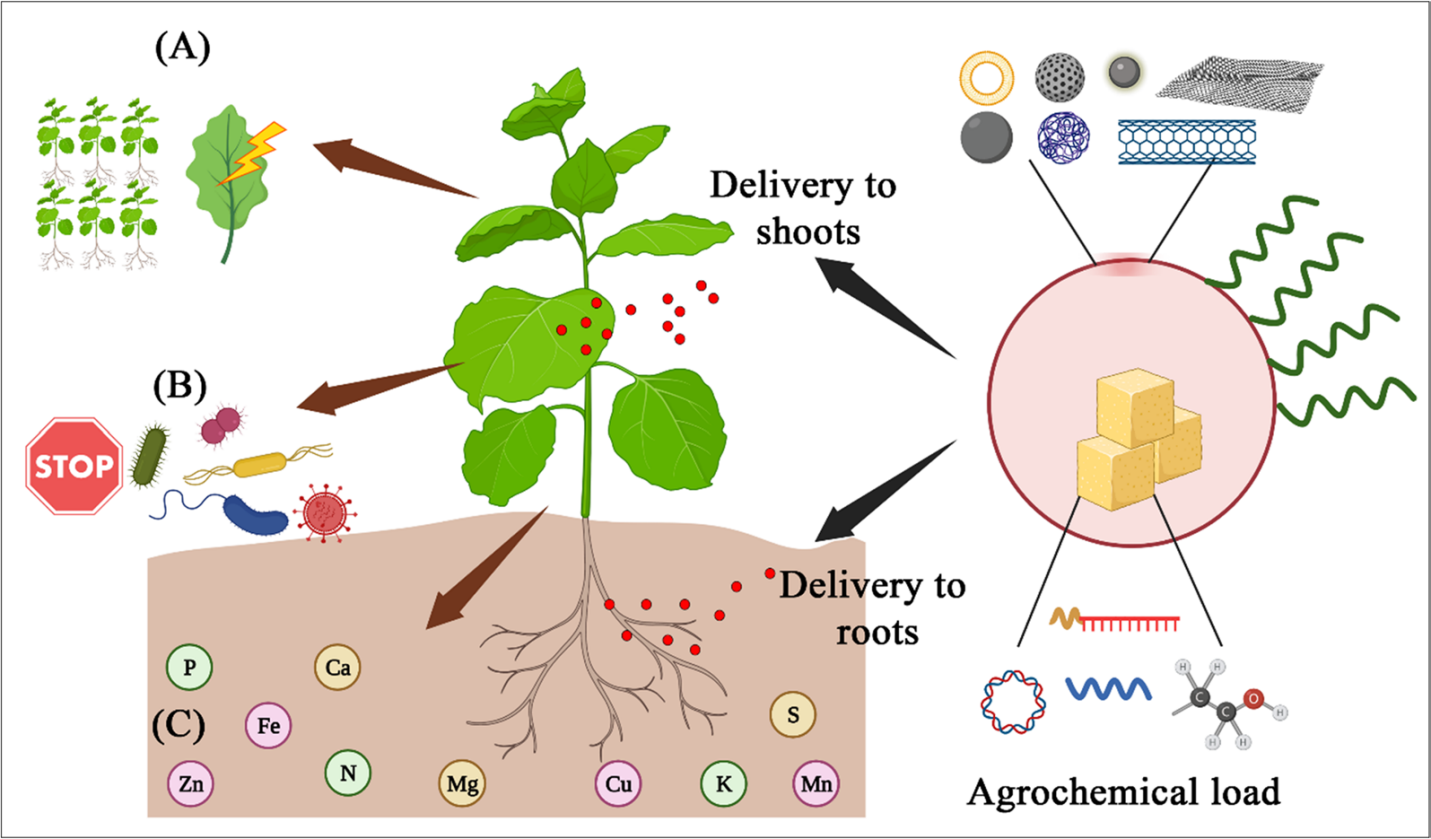
Nanoparticles enable the delivery of agrochemicals (peptides, nucleic acids, small molecules) through diverse nanomaterial systems (liposomes, silica/polymeric/metallic nanoparticles, carbon nanotubes, graphene nanosheets, quantum dots). The nanomaterials can be used with or without surface tethering for specific targeting moiety (green wavy line in the figure) in order to **A**; improve the nutritional quality and yield of economically important crops, **B**; cure plant diseases, and **C**; improve nutrient level, fertility, and soil health

In the wake of the overgrowing population in the world and the burden on the food supply through agriculture, farmers are using fertilizers, pesticides, and herbicides in excessive amounts in order to improve agricultural yield, crop quality, and nutritional value. Excessive use of such agrochemicals has reduced soil health and threatened the crop-associated ecosystem because of their carcinogenicity and/or mutagenicity [86]. Hence, it is imperative to find alternate ways to improve agriculture yield while maintaining soil health. Nanoparticulate systems offer a tremendous advantage in this regard. As mentioned earlier, the NPs can be used for targeted delivery of nutrients, antibiotics, fertilizers, pesticides, and herbicides via various routes such as seed priming, foliar application, trunk injection, spraying on shoots, or mixing in soil (uptake via roots). Figure 9 showing the implementation of nanotechnology to supplement Mg and Fe deficiencies in tomato plants improved plant nutrition and quality. Apart from size, surface charge, and surface area to volume ratio, the NPs offer excellent advantages of target specificity and controlled release of biomolecules. Due to these two properties, the NPs deliver the biomolecules/agrochemicals in the spatiotemporal way i.e. at exact place (e.g. cell or cell organelle) at exact rate and in the exact amount. Various types of biomolecules delivered via NPs have been shown to produce beneficial effects on crops as well as on soil (Fig. 10). For example, Karny et al. synthesized liposomes from a plant-derived lipid-hydrogenated L-α-phosphatidylcholine (isolated from soybean) encapsulating nutritional supplements like iron (Fe) and magnesium (Mg). Upon spraying on leaves, the authors showed that liposomes having size of 88.37 ± 21.13 nm traveled in the bidirectional way and reached other leaves as well as roots within 24 h with maximum intensity reaching within 72 h. The delivery of nutritional supplements of Fe and Mg via these liposomes led to the recovery of plants from chlorosis (reduced production of chloroplast) as well as epinasty (downward and outward growth of plant due to differential and reduced growth rates) in tomato plants within 14 days after treatment. The turgor pressure was also restored in plants upon liposomal treatment as compared to the application of nutritional supplements in non-NPs form [87]. In another report, Bao et al. used layered double hydroxide sheet formed NPs (LDHNP) to deliver single-stranded DNA (ssDNA) to plant cells. They achieved the association of negative charged DNA to positively charged LDHNP via electrostatic adsorption. Using 5-day-old seedlings of *Arabidopsis thaliana* and *Nicotiana tobacum* cv Bright Yellow 2 (BY-2) suspension cells, the efficacy of ssDNA-LDHNP was tested. The authors observed that the NPs gets internalized in cell nuclei within 60 min, making them a potential candidate for novel gene delivery [88]. On similar grounds, Mitter et al., showed that double stranded RNA and RNAi can be delivered to *Nicotiana tobacum* via LDHNPs for their protection against pepper mild mottle virus (PMMoV) and cucumber mosaic virus (CMV). Tremendous stability and durability to adhere on leaf surface for 30 days after a single spray, and passive/active internalization to gain entry into cells of unsprayed new leaves were key characteristics of their formulation. Furthermore the dsRNA and RNAi loaded LDHNP protected plant for 20 days from PMMoV and CMV challenge (given on 5th day) proving excellent antiviral activity of NPs in plants [89]. In an interesting report, Santana et al. developed ~ 5 nm cadmium quantum dots (Cd-QDs) covalently linked with β-cyclodextrin and a chloroplast targeting peptide (RbcS-peptide). Authors demonstrated that due to presence of RbcS-peptide, almost 70–80% of chloroplast contained QDs after injection into abaxial side of leaves. Owing bucket like structure of β-cyclodextrin, authors showed that agrochemicals like methyl viologen (a herbicide) and ascorbic acid (a plant metabolite and vitamin) can be delivered specifically to chloroplast. As compared to chemicals without nanoparticles, the QDs loaded with methyl viologen produced higher ROS, whereas ascorbic acid loaded QDs acted as scavenger and reduced the ROS level significantly. This reports is direct proof of delivery of agrochemical in very controlled fashion to particular cell organelle (i.e. chloroplast) [91]. Thagun et al. used different approach by combining cell penetrating peptides (CPPs) with plasmid DNA or siRNA to form globular complexes having sub 100 nm size. Owing to cationic charge of CPP and resulting nanocomplexes with plasmid DNA/siRNA, upon foliar spraying, the complexes entered and localized into the epidermal cells of Arabidopsis and tomato leaves. In case of plasmid DNA having β-glucoronidase reporter gene (GUS), the CPP-DNA complexes improved expression of this gene and activity of GUS as compared to control counterparts (only plasmid DNA and only cell CPP). Furthermore, the CPP-siRNA complexes, after internalization silenced expression of fluorescent proteins. Additionally, using chloroplast targeting peptide along with CPP-DNA/siRNA complex, authors showed that the expression of luciferase gene and luciferase activity, as well as silencing of fluorescent protein expression, can be successfully altered in the chloroplast of Arabidopsis leaves. These remarkable results suggest that in the case of the economically important plants, the crop quality and crop yield can be manipulated by specific gene targeting in a more controlled way [92]. Carbon NMs has gained wide attention due to their remarkable physicochemical properties. Just like other materials, they can also be used for delivering agrochemicals. For example, Kabiri et al. prepared cube-shaped micronutrients (Copper (Cu) and Zinc (Zn)) loaded graphene oxide (GO) fertilizers. Upon dissolution study, authors found out that these GO based fertilizers release micronutrients in both immediate (~ 30% release within 5 h) as well as a sustained way (~ 80% release over the period of 3 days) just as needed for the seed germination and plant growth. After treating the wheat crop (*Triticum durum cv. Yallaroi*) with micronutrient loaded GO fertilizers, the micronutrient uptake was significantly higher compared to only chemicals. The grain dry mass of wheat was significantly higher for soil treated with GO fertilizers as compared to non-treated and chemically treated soil [93]. Similarly, Pyridaben (Pyr), chlorpyrifos (Chl) and beta-cyfluthrin (Cyf) loaded GO nanocomposites provided protection against spider mites in greenhouse-grown bean plants. Authors observed significantly less LD50 values for GO-pesticide nanocomposites as compared to pesticides alone suggesting the synergistic activity of GO and pesticides against *Tetranychus truncates* and *Tetranychus urticae*. In greenhouse-grown bean plants, these nanocomposites showed almost 80% mortality indicating their potent efficacy [94]. Just like CPP-siRNA complexes, single-walled carbon nanotubes (SWNTs) were also found to be effective in loading and delivering siRNA for gene knockdown in *Nicotiana benthamiana* plants. Upon administration on abaxial sides of leaves and incubation for 6 h, almost 70–80% siRNA-SWNTs were successfully internalized. After internalizing, those siRNA-loaded SWNTs showed silencing of GFP expression for a shorter duration (3 days). The silencing action was abolished after longer exposure (7 days) due to the degradation of siRNA. Yet, the SWNTs offered stability to siRNA and showed 12 h increase in its residence time inside the plant cells as compared to naked siRNA. In case of polymeric NPs, Fischer et al. have shown use of smart enzyme responsive lignin NPs for delivery of fungicide- Pyraclostrobin against worldwide grapevine trunk disease- esca. Esca associated fungi, tracheomycotic *Phaeomoniella chlamydospora* (Pch) and *Phaeoacremonium minimum* (Pmi) secret enzymes like laccase and peroxidases. These enzymes degrade lignin NPs that leads to release of pyraclostrobin in controlled way depending on the degradation rate of NPs. The authors showed that lignin nanocarriers had excellent stability in biological fluid such as wood extract and showed no fungicide leakage during storage period. Upon injecting single dose of pyraclostrobin-loaded lignin NPs in the trunk of the plants, they observed significant reduction in esca symptoms over the period of 1 year. For next 3 years, no additional symptoms were developed, which proved that the fungicidal NPs had potent action. This study is an excellent example of field study of fungicidal NPs for long period of 5 + years. Additional field trials conducted by authors showed better antifungal activity against esca as compared to commercial product: F500 containing 6 mg/mL pyraclostrobin [95]. Apart from aforementioned NMs (QDs, liposomes, polymeric nanoparticles, peptides-biomolecules nanocomplexes), the metal nanoparticles have also shown strong promises in the field of agrochemical delivery to important crops. Cai et al. used Fe_3_O_4_NPs using foliar spray in *Nicotiana benthamiana* plants. The authors gave compelling evidence for accumulation of Fe_3_O_4_NPs in all parts of the plant except roots. Unlike few reports mentioned previously, for these nanoparticles foliar application method led to significant uptake and transport as observed by the authors with transmission electron microscopy and elemental analysis of different parts of plants. Moreover, the fresh and dry weight of plants as well as the phytohormone levels were significantly improved after Fe_3_O_4_NPs treatment. One such phytohormone-salicylic acid (SA) is involved in response to a stress like viral/bacterial/fungal infection, salt change, temperature changes, and water level changes. Authors showed that SA level was significantly high in NPs treated tobacco plants. Thus, disease induction after tobacco mosaic virus (TMV) inoculation was low in case of Fe_3_O_4_NPs-treated plants as compared to untreated group. Thus, these metal nanoparticles acted as nanofertilizers providing iron supplement along with antiviral agents against TMV [96]. Young et al. have shown the usage of ZnO-Cu-Si gel composite as an antibacterial material for treatment of citrus plant infection caused by common phytopathogen *Xanthomonas citri*. This nanocomposite exhibited ~ tenfold stronger antimicrobial activity (MIC 15–30 µg/mL) against phytopathogen as compared to control counterparts Cu salts, ZnO, Si gels etc.). Moreover, with very low phytotoxicity, these nanocomposites showed remarkable reduction in citrus canker incidence as compared to untreated group. In the untreated plants, the canker incidence was as 25%. The disease legions and severity were significantly lower after the treatment with nanocomposites. Here, in this nanosystem, the Cu and Zn supplements delivered by the Si gel offered antimicrobial action through protein inactivation, DNA damage, and oxidative stress to phytopathogen [97]. Shayganfar and Akhzari reported a general non-specific response by three Thymus plants after exposure to different-sized AgNPs. The essential oil contents were elevated after stress offered by AgNPs, but interestingly, this stress response was highly dependent on the size and concentration of NPs as well as on the species of the plant under study. This highlights the variable effects of metal NPs [98]. Iannone et al. reported the growth-stimulating effect of Fe_3_O_4_NPs in soybean and alfalfa plants. These NPs improved chlorophyll contents and led to an increment in root and shoot length indicating a positive stimulus in plant growth. Furthermore, the citric acid coating on these NPs offered excellent stability and compatibility. Hence, these Fe3O_4_NPs did not show any oxidative damage or cell death [99].

## Limitations and future prospects

**Fig. 11:**
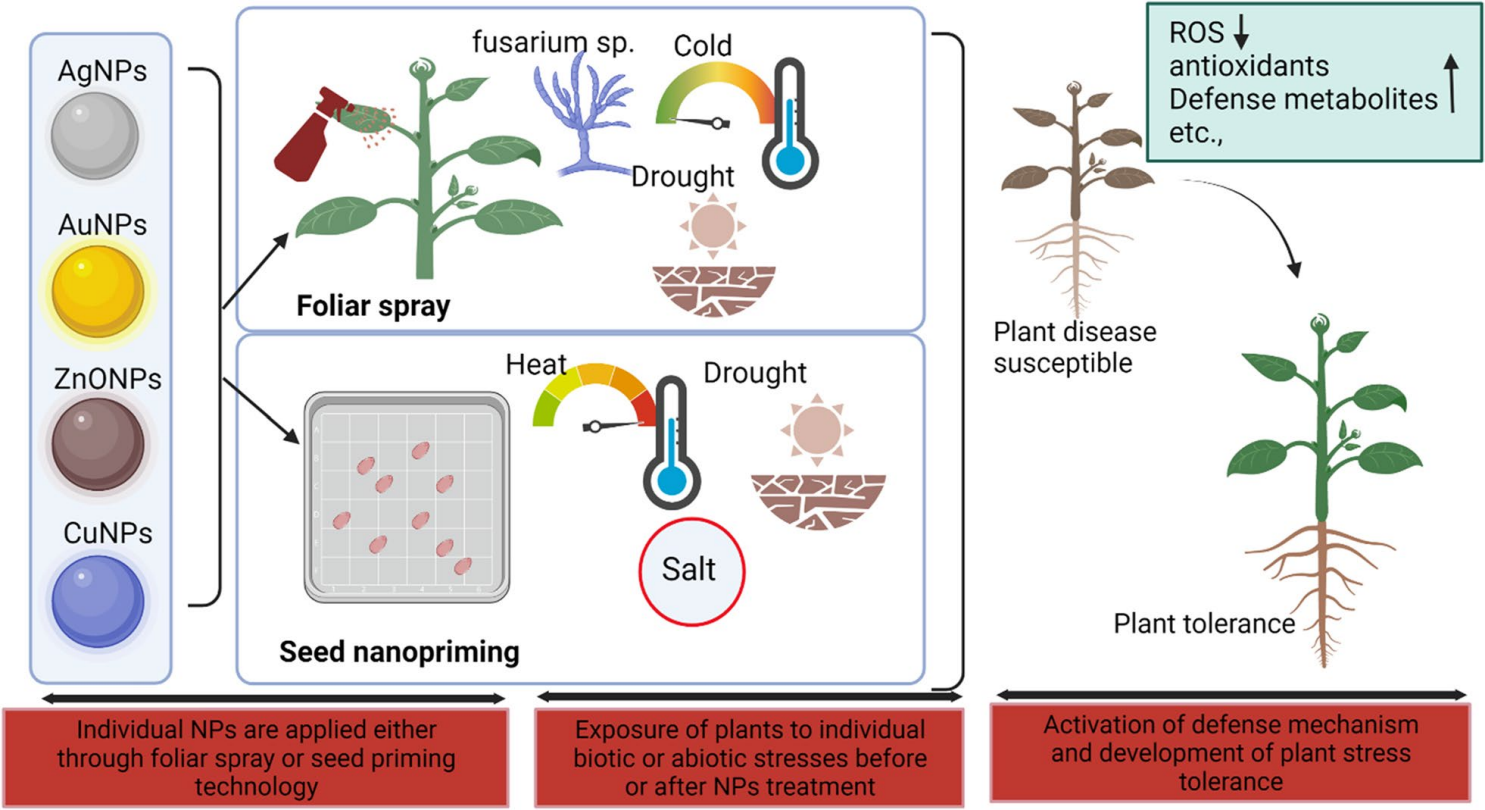
Beneficial NPs application methods for exploring plant tolerance mechanisms against biotic sand abiotic stresses. Based on the references covered in this review, this figure was created using biorender software https://biorender.com/

Just like every other technology, the nanotechnological means intended for an application in agriculture field suffers with advantageous (Fig. 11) and some drawbacks. Among different countries located around the world, only European Union and Switzerland incorporated nanoparticle specific provisions in legislation for agriculture. While non-EU countries are still indirectly dependent on the guidance of industries due to lack of nanoparticle specific provisions in their current legislations [100]. In the wake of fast-growing nanotechnology field and its application in this field, the unified policies and regulations regarding the synthesis of nanomaterials, use of nanomaterials, and their removal/elimination from the environment must be prepared and updated constantly as per the current state of the art nanotechnology. The practice of NPs in agriculture is still in the preliminary stage, and their full potency in agriculture is gradually transferring from the theoretical knowledge to the field application. For this, the researchers and industries face usual challenges, including high processing costs, standardizing the research protocols, concerns about public health and the environment, etc. The green synthesis methods from cheaper materials like copper, zinc, iron solve this problem temporarily. Yet, efforts must be taken to reduce the cost of final nanotechnological product so that it can be made available for large scale agricultural use by the farmers. From this review, we realized that the different types of NPs synthesized using green methods, and different methods of NPs application produce positive effects in plants. However, there is no accurate evidence that specific application method and specific type of NPs in agriculture is completely safe for plant and soil health. Therefore, it is indeed necessary to explore these gaps in knowledge. In general, that smaller sized nanoparticles at lower concentrations improve the physiological/biochemical status of plants under stress and non-stress conditions (Fig. 8) via seed priming & foliar spray methods only when used for a short duration. The use of nanoparticles for long duration and its impact on crop-associated food chain is not yet fully understood. NPs toxicity is a very important benchmark for transferring the technology from lab to field. To minimize the nanotoxicity, researchers have coated nanoparticles with different moieties, but, at present, no comprehensive report is available that covers both short-term and long-term effects of nanoparticles on crops and crop-assisted food chain. These limitations should be covered in each study as the nanotoxicity study may vary depending on type of nanomaterial, crop species, and agricultural conditions. Special efforts can be driven to assess any possible resistance and defensive mechanism developed by plants to the action of these nanoparticles upon excessive or long-term usage. Soil fertility is another mission to be achieved while using NPs in agriculture because fertile soil contains all essential nutrients and millions of microbes that regulate plant growth in a healthy way. Therefore, soil health should be monitored for an extended duration after using these nanoparticles to ensure that it does not affect soil fertility regardless of the method of NPs application. Despite all these concerns, nanoparticles serve as a novel and promising tool in the agricultural field.

## Conclusion and future prospects

A vast literature is published regarding the usage of metallic nanoparticles in agriculture. Researchers have reported various types of metal nanoparticles (Cu, Ag, Au etc.) ranging from X to Y nm (This could be a general range from all cited papers in this review articles) and having various shapes such as spherical, triangular, cubic etc., for the improvement of crop/plant performance under stress and normal condition. These nanoparticles were applied to plants at laboratory scale as well as field scale by many application methods. However, it is necessary to select the most suitable method and nano formulation to achieve optimal sustainability in agriculture. To answer this question, it is necessary to review and compare the state-of-the-art methods as well as their outcomes. Therefore, we have attempted to fill this gap where we specifically pointed out the implementation of smaller sized, and lower concentration of nanoparticles; seed priming and foliar spray technology to plants as a safer method that minimize toxicity, exhibited better plant performance during stress and non-stressed conditions. Additionally, drawing conclusions from literature review, our article also put forward an urgent issue of regulatory aspects to control use of nanotechnology in agriculture field. Further, we summarized the use of nanomaterials to deliver biomolecules as an alternative strategy to prevent the use of chemical fertilizers and also could be adapted as a safer and cleaner technology for sustainable agriculture.

Nanotechnology could also have beneficial impacts on soil health if used in regulated ways. Furthermore, the agro-industries relying on improved farming could perform in a better way to produce food while coping up with increasing population and food demand globally. In the wake of reduced farming land and overgrowing population across the globe, nanoparticle-mediated improved agriculture certainly seems a fantastic way towards better future. However, the path towards it has many hurdles, such as nanotoxicity in the ecosystem, detrimental environmental impact such as poor soil health, and a lack of proper authoritative regulatory bodies to control the use of nanomaterials. Once these barriers are crossed, the nanoparticles could offer an excellent way for sustainable green agriculture.

Overall, our review helps the researchers in this field to select best possible nano- formulation in terms of synthesis methods and physicochemical properties and also suggested the most suitable method for applying nano-formulation in agriculture that resulted in least to no phytotoxic effects. Our article also points out what is still lacking in this field; e.g. (i) long term effects of application of nanotechnology in agriculture field, (ii) regulatory aspects. So, in a way our efforts to put this altogether can become a guidance for researchers in this field.

## Data Availability

Datas are available upon reasonable request.
